# Evolutionary insights from *de nov*o transcriptome assembly and SNP discovery in California white oaks

**DOI:** 10.1186/s12864-015-1761-4

**Published:** 2015-07-28

**Authors:** Shawn J. Cokus, Paul F. Gugger, Victoria L. Sork

**Affiliations:** Molecular, Cell, and Developmental Biology, University of California, 3000 Terasaki Life Sciences Building, 610 Charles E. Young Drive East, Los Angeles, CA 90095-7239 USA; Ecology and Evolutionary Biology, University of California, 4140 Terasaki Life Sciences Building, 610 Charles E. Young Drive East, Los Angeles, CA 90095-7239 USA; Institute of the Environment and Sustainability, University of California, 300 La Kretz Hall, 619 Charles E. Young Drive East, Los Angeles, CA 90095-1496 USA

**Keywords:** Annotation, *De novo* assembly, Divergence, *d*_N_/*d*_S_, *Quercus douglasii*, *Quercus garryana*, *Quercus lobata*, RNA-Seq, Single-nucleotide polymorphism, Transcriptome

## Abstract

**Background:**

Reference transcriptomes provide valuable resources for understanding evolution within and among species. We *de novo* assembled and annotated a reference transcriptome for *Quercus lobata* and *Q. garryana* and identified single-nucleotide polymorphisms (SNPs) to provide resources for forest genomicists studying this ecologically and economically important genus. We further performed preliminary analyses of genes important in interspecific divergent (positive) selection that might explain ecological differences among species, estimating rates of nonsynonymous to synonymous substitutions (*d*_N_/*d*_S_) and Fay and Wu’s *H*. Functional classes of genes were tested for unusually high *d*_N_/*d*_S_ or low *H* consistent with divergent positive selection.

**Results:**

Our draft transcriptome is among the most complete for oaks, including 83,644 contigs (23,329 ≥ 1 kbp), 14,898 complete and 13,778 partial gene models, and functional annotations for 9,431 *Arabidopsis* orthologs and 19,365 contigs with Pfam hits. We identified 1.7 million possible sequence variants including 1.1 million high-quality diallelic SNPs — among the largest sets identified in any tree. 11 of 18 functional categories with significantly elevated *d*_N_/*d*_S_ are involved in disease response, including 50+ genes with *d*_N_/*d*_S_ > 1. Other high-*d*_N_/*d*_S_ genes are involved in biotic response, flowering and growth, or regulatory processes. In contrast, median *d*_N_/*d*_S_ was low (0.22), suggesting that purifying selection influences most genes. No functional categories have unusually low *H*.

**Conclusions:**

These results offer preliminary support for the hypothesis that divergent selection at pathogen resistance are important factors in species divergence in these hybridizing California oaks. Our transcriptome provides a solid foundation for future studies of gene expression, natural selection, and speciation in *Quercus*.

**Electronic supplementary material:**

The online version of this article (doi:10.1186/s12864-015-1761-4) contains supplementary material, which is available to authorized users.

## Background

Transcriptome sequences provide valuable resources for research in comparative genomics, population genetics, and evolutionary biology. Although numerous crop and some tree species have fully sequenced and annotated reference transcriptomes [1–3], there is still a need for more sequences from ecologically important taxa. Oaks (*Quercus* spp.) are ecologically and economically important species that lack a reference genome or transcriptome and would benefit from such resources [4]. To date, the genomic sequence resources available in oaks are limited primarily to expressed sequence tags (ESTs) in the European white oaks, *Q. robur* L. and *Q. petraea* (Matt.) Liebl. [5–8], and in the eastern North American oaks, *Q. alba* L. and *Q. rubra* L. [9], and many of those resources remain under development [4]. Research in population genetics, genomics, evolutionary biology, hybridization, response to the environment, and global change biology of oaks would benefit from an annotated transcriptome assembly and deep panel of nucleotide polymorphisms, especially in species and geographic regions with fewer such resources to date. High-throughput, short-read RNA-Seq data have enabled the rapid development of reference sequences and identification of single-nucleotide polymorphisms (SNPs) among those sequences [10, 11].

Here, we present the most complete transcriptome for white oaks (*Quercus* section *Quercus*) and among the largest SNP data sets available for trees using pooled RNA-Seq data from 22 *Q. lobata* Née (valley oak), 1 *Q. garryana* Dougl. (Oregon white oak), and — accidentally — 1 probable hybrid *Q. lobata* × *Q. douglasii* Hook. & Arn. (blue oak) (Table 1). Using multiple species and multiple individuals within *Q. lobata* enabled us to identify large SNP panels useful for both inter- and intraspecific studies, as well as develop reference sequences relevant to a broader array of California white oaks. These species are closely related [12] and hybridize with varying frequency in zones of overlapping distribution [13–15]. Each has distinct morphological characteristics and ecological niches, with *Q. lobata* occupying valley floors, *Q. douglasii* occupying hillsides, and *Q. garryana* occupying wetter, higher elevation sites.

**Table 1:** Sample information

Species	Site name	Sample number	Tissue	Latitude (°)	Longitude (°)	Elevation (m)
*Quercus lobata*	Bradley	2	small leaf	35.86508	−120.80903	157
*Q. garryana*	Branscomb	1	unopened buds	39.64312	−123.53139	590
*Q. lobata*	Diamond Springs	3	unopened/opening buds	38.68972	−120.83063	532
*Q. lobata*	El Dorado	4	smallest leaf	38.6727	−120.85180	491
*Q. lobata*	Fort Tejon	1	small leaf	34.87476	−118.89410	994
*Q. lobata*	Fort Tejon	6	male flower/leaf opening bud	34.8743	−118.89538	994
*Q. lobata*	Hastings	163	smallest leaf	36.38751	−121.54992	540
*Q. lobata*	Hastings	247	small leaf	36.38061	−121.55290	634
*Q. lobata*	Laytonville	2	unopened buds	39.68847	−123.48866	477
*Q. lobata*	Malibu Creek	1	male flower opening	34.10143	−118.71223	192
*Q. lobata*	Malibu Creek	3	male flower opening	34.10102	−118.71203	192
*Q. lobata*	Mariposa	2	smallest leaf	37.46107	−119.87966	618
*Q. lobata*	Mariposa	3	male flower/small leaf opening bud	37.46038	−119.87327	618
*Q. lobata*	McLaughlin	1	smallest leaf	38.8717	−122.42200	651
*Q. lobata*	McLaughlin	2	smallest leaf	38.8717	−122.42640	646
*Q. lobata*	Mt. Diablo	5	small leaf	37.90195	−121.99319	105
*Q. lobata*	Mt. Diablo	1_2	expanding male flower; small/medium leaf	37.88025	−121.96494	260
*Q. lobata*	Oneals	1	smallest leaf	37.15651	−119.73781	355
*Q. lobata*	Sedgwick	32	smallest leaf	34.70143	−120.04046	349
*Q. lobata*	Sedgwick	663	male flower/small leaf opening bud	34.68894	−120.03593	332
*Q. lobata*	Springville	5	full-size young leaf	36.09927	−118.86832	217
*Q. lobata–douglasii* hybrid	Springville	1	full-size young leaf	36.07971	−118.89890	233
*Q. lobata*	Woodson	2	small leaf	39.90998	−122.08987	66
*Q. lobata*	Woodson	6	small leaf	39.91216	−122.08814	66

We used our reference transcriptome and SNP data to perform preliminary comparative analyses, testing for signatures of natural selection that can provide insight into the factors that explain phenotypic or ecological differences among species. A number of approaches have been employed in the literature to understand species divergence, including phenotypic selection experiments, quantitative trait locus studies, and genome-wide sequencing [16–18]. Together, they strongly implicate natural selection, not just genetic drift, as a prominent force in speciation and divergence. However, it is more complicated to explain the maintenance of species boundaries and ecological specialization in species that naturally hybridize. For example, even modest hybridization can break down species boundaries and lead to homogenization [19] or maladaptation [20], or it could facilitate the transfer of adaptive alleles among species [21], among other possibilities [22, 23]. One hypothesized explanation for the persistence of morphological and ecological distinctions among species despite hybridization is divergent natural selection at ecologically relevant genes, such as those involved in biotic or abiotic stress responses [24]. With current large-scale single-nucleotide polymorphism (SNP) data sets among closely related species and classical molecular tests for selection, we now have the tools to test this hypothesis by revealing specific genes and functional classes of genes that are under divergent selection among lineages [25, 26].

The genus *Quercus* (oak) has long been recognized for its propensity to hybridize yet maintain ecological and morphological differentiation [27], which has led to the proposal of the ecological species concept [28]. Although pre-zygotic isolating mechanisms exist [29, 30], oaks frequently hybridize and species maintenance must be explained by other factors related to divergent selection in many cases [31, 32]. Our preliminary analyses identify genes under divergent selection among several California white oaks to test the hypothesis that selection by abiotic and biotic stresses contribute to ecological divergence and maintenance of oak species boundaries. We used our transcriptome-wide SNPs to estimate the ratio of nonsynonymous to synonymous substitution rates (*d*_N_/*d*_S_) as evidence for divergent (>1) or purifying (<1) selection [26], and Fay and Wu’s *H* as evidence for positive selection (<0). In particular, we tested for functional classes of genes based on Pfam annotations [33, 34] that showed the strongest evidence of divergent or positive selection among these ecologically distinct species.

## Results and discussion

### Transcriptome assembly

Approximately 12–22 million 100-base paired-end reads per individual (*n* = 24) and 420 million read pairs total (84 Gb) were obtained (NCBI: PRJNA282155). Adapter contamination was minimal (≈1 in 5,000 reads with a possible fragment at any position, and, of these, mostly reads entirely rather than partially adapter). The insert size distribution was short enough that 237 million read pairs overlapped unambiguously and thus were merged, forming 35 Gb of virtual single-end reads of 100–184 bases. The Ray *de novo* assembler [35] was used to assemble these and unmerged paired ends as a pool into a draft transcriptome of 88,595 preliminary contigs of sizes 203–16,982 bp totaling 73 Mbp (N50 = 1.2 kbp). Approximately 23,000 contigs of total 41 Mbp were of size ≥ 1 kbp, and a large number of the remaining contigs were quite short and of low coverage (Additional file 1) and may be (fragments of) low-expression genes, intron leakage (as can be common in some RNA-Seq preparations), 5ʹ- and 3ʹ-UTR extremes that tail off to low abundance or are shared by multiple genes, etc.

Thousands of contig pairs were found to share intervals of ≥ 35 bp of exactly identical sequence away from regions masked by Tandem Repeats Finder [36]. Pairs of contigs whose ratio of average coverage differed by no more than 2:1 and that had only one uniquely aligning interval compatible with end-to-end joining were joined. Thus, some 9.6 k contigs of total ~8 Mbp were joined and replaced with ~4.6 k larger contigs of total ~7 Mbp. These joined contigs were found to be much enriched for compatibility with amino acid-coding open reading frames (ORFs), such that often one model missing its 3ʹ-end was joined with one missing its 5ʹ-end. An effort was also made to identify over-merged contigs and split them, although a survey of likely coding regions suggested that over-joining was not prevalent (see “Gene models and UTRs” below).

The final number of contigs after merging and splitting was 83,644 (N50 = 1.2 kbp) (see http://genomes.mcdb.ucla.edu/OakTSA/ for files containing assembled contigs, all annotations, variant calls, and other results discussed below). The resulting 72.5 Mbp draft transcriptome is nearly 10 % of the total estimated oak genome sequence of 750 Mbp [37] and is comparable to, although somewhat larger than, the *Populus trichocarpa* [1] and *Arabidopsis* [38, 39] transcriptomes.

### Assembly quality

Our transcriptome assembly is of high quality and purity. First, almost all 100-mers in almost all transcriptome contigs are highly unique (Additional file 2). Thus, even though *Quercus* has high genetic variation [40–42], it does not appear that many genic loci were multiply assembled (e.g., forming separate contigs from divergent alleles or splice variants). Second, we found that 70–78 % of original read ends per individual mapped back to our assembled reference with mapping confidence ≥ 99 % (or 80–89 % of reads with any mapping quality), comparable to typical RNA-Seq experiments with established references. Third, the C+G content (mean 39 %) is consistent with that of other oaks [37] and plants in general [38, 43]. The distribution of C+G content across coding sequences, 5ʹ-UTRs, and 3ʹ-UTRs is distinct and descending across these groups, and are similar to *Arabidopsis* (Additional file 3). The oak transcriptome contigs lacking gene models, which are generally short and of low expression, have low C+G content similar to 3ʹ-UTRs, consistent with being enriched for introns. In *Arabidopsis*, introns also have low C+G content similar to 3ʹ-UTRs [39].

Fourth, *Quercus* is by far the dominant organism represented in the contigs and non-oak contamination is not prominent, both as determined by comparisons to known sequences (see “Gene models and UTRs”), as well as the 18 contigs putatively identified as rRNA used as indicators. One rRNA contig had high-coverage, high-identity best match to the *Quercus rubra* chloroplast, one to the *Gossypium hirsutum* (cotton) mitochondrion (note that NCBI does not have a full *Quercus* mitochondrial sequence but only a small fragment), and the rest to bacterial sequences. However, of the 2.2 million original reads that aligned to these known sequences, 87 % matched the *Q. rubra* chloroplast, 11 % matched the cotton mitochondrion, and just 2 % matched the bacterial sequences. Furthermore, percent identity tended to be about 99 % with the chloroplast or mitochondrial reads and considerably lower with the bacterial reads.

Additionally, our Californian oak (*Q. lobata*/*garryana/douglasii*) transcriptome contained many contigs in common with European *Q. robur* EST data and the eastern North American *Q. alba* 454 EST data (Additional file 4), although our assembly consisted of more total contigs with twice the mean and maximum length (Additional file 5). For example, 55 % of our California *Quercus* contigs were found in *Q. robur* ESTs, and 19 % were found in *Q. alba* ESTs. In contrast, the proportions of *Q. robur* (62 %) and *Q. alba* (79 %) found in our *Quercus* transcriptome are higher. Together, these reciprocal comparisons indicate that the *Q. alba* dataset is largely found within our *Quercus* or the *Q. robur* datasets, whereas the *Q. robur* and our *Quercus* datasets each contain many unique transcripts.

Further indications of transcriptome quality and purity appear below.

### Repeats and transposons

Only 4.4 % of the transcriptome contig base pairs are marked as repetitive by RepeatMasker, breaking down as 1 % long interspersed elements (LINEs, mostly L1/CIN4 and RTE/Bov-B), 0.9 % Ty1-copia and gypsy/DIRS1 long terminal repeats (LTRs), 0.5 % DNA transposons (0.2 % hobo-Activator), 0.5 % unclassified, 0.7 % low complexity, 0.5 % simple repeats, and 0.1 % small RNA. This overall percentage is comparable to a 454-based transcriptome assembly in *Pinus contorta*, but much higher than that observed in many other EST-based studies in plants [44]. LINE/LTR/DNA-transposon containing contigs tended to be higher than average coverage, thus the large pool of low expression contigs (Additional file 1) does not seem to be mostly from transposons or retroelements.

### Gene models and UTRs

About 4.4 k high-confidence, complete, single-exon gene models and 101 intron-containing models were selected from a preliminary GlimmerHMM [45] calling with *Arabidopsis* parameters to bootstrap the AUGUSTUS gene caller [46]. After re-training and re-running AUGUSTUS, we explored coverage patterns in contigs containing tentative gene models. A random sampling of contigs as well as most of the 384 contigs that had more than one gene model showed expected coverage patterns. However, 90 contigs with multiple gene models showed sharp coverage discontinuities between models, suggesting such contigs were over-assembled fusions of multiple transcripts. These were split into separate contigs and final AUGUSTUS runs made. The result is 14,898 complete gene models, 6,826 missing the 3′ end, 4,577 missing the 5′ end, and 2,375 internal fragments (missing both 3′ and 5′ ends). All models total 28 M coding nucleotides. Twenty-four percent of all models and nine percent of complete models contain introns, and only one percent of contigs with at least one gene model have multiple gene models. Assuming that the 13,778 partial gene models represent at least 5,000 separate genes, then about 20,000 total genes are represented in our bud/leaf/flower transcriptome — a number comparable to what one would expect from a given tissue at a given life stage in *Arabidopsis* (e.g., NCBI SRX145413 Col-0 wild type leaf RNA-Seq as a generic representative [47]).

The amino acid usage and model length distributions compared favorably with *A. thaliana* TAIR10 genes [38] (Additional file 6). 5ʹ- and 3ʹ-UTR lengths are reasonable, amounting to about 1/3 of transcript lengths, but are slightly longer than in *Arabidopsis* (Additional file 6), perhaps due to the tendency for model organism projects to be somewhat conservative in UTR annotation and *Quercus* having a genome roughly six times the size of *Arabidopsis* [37]. Our oak draft transcriptome has 88 % of the complete protein count and 94 % of the partial protein count of core eukaryotic genes (CEGMA 2.4 [48]) found in *Populus trichocarpa*, with 69–74 % of genes having orthologs. Finally, an analysis based on reciprocal best hits with BLASTp alignments between six-frame contig translations and a large pool of NCBI proteins showed that the most common organisms closest to individual contigs of a draft version of the transcriptome were all plants, especially (in descending order) *Vitis vinifera*, *Populus trichocarpa*, *Glycine max*, *Arabidopsis thaliana*, *Ricinus communis*, and *Medicago truncatula*.

### Gene and domain annotation

We identified 9,431 oak–*Arabidopsis* gene pairs as being one-to-one orthologs (7,543 with a complete oak model). These were generally near-entire on both sides, with a mode of 70 % amino acid identity (Additional file 7). Oak contigs of low coverage and *Arabidopsis* loci of low expression (based on accession SRX145413 [47] as a generic RNA-Seq data set) are much less likely to have an orthologous pairing, but many of those at higher coverage levels are captured (Additional file 8). Additionally, 50 % of *Arabidopsis* SRX145413 reads aligning to an *Arabidopsis* gene are to genes with a called oak ortholog, suggesting the ortholog calls capture many commonly expressed genes. Further, gene expression levels of orthologous pairs are correlated (Additional file 9). In addition, the distribution of Gene Ontology (GO) Plant Slim [49] terms is comparable between all TAIR10 loci and those having a called *Quercus* ortholog (Additional file 10), demonstrating that the *Quercus* transcriptome contains a representative and wide variety of genes. Additional functional annotation is provided by ~50,000 hits to Pfam from 19,365 distinct oak gene models, with ~3,800 of the 14,831 Pfam accessions appearing at least once. These numbers are similar to a run on *Arabidopsis* TAIR10 with the same parameters, resulting in ~48,000 hits to ~22,000 genes (collapsing over splice variants, giving for each gene and domain the maximum number of hits over the gene’s versions) with ~4,200 distinct Pfam accessions appearing at least once.

### Variant calls

Using a GATK-based pipeline [50], we found ~1.7 million possible sequence variants when considering all 24 samples (*Q. lobata*, *Q garryana*, and the *Q. lobata*–*Q. douglasii* hybrid). After filtering and restricting only to SNPs, we found over 1.1 million, of which 98.5 % were diallelic, 1.5 % were triallelic, and 0.02 % were tetrallelic, with overall transition:transversion ratio of 1.9. The filtered diallelic SNP loci were used in subsequent analyses below. Among only the 22 *Q. lobata* samples, we identified about 900 k diallelic SNPs. Our SNP data set is the largest available in any *Quercus* species [5] and among the largest for trees [51–53].

The overall SNP locus rate per base pair was 1.5 % among all samples and 1.2 % within *Q. lobata*. Such rates vary depending on organism, degree of sampling from a population, and depth of sequencing, but those here are generally consistent with studies in other plants [54, 55], including oaks (when considering the rate across all contigs, not just contigs with variant loci) [5, 41], but somewhat lower than *Arabidopsis* that was sampled more broadly [56]. SNP locus rates varied in patterns similar to those in an *Arabidopsis* genomic study [56] depending on whether the loci were within coding, intron, or UTR sequence (Additional file 11). The main difference seen can be explained by a drop in our power to detect variants at contig edges where coverage drops off (whereas transcript edges are not special when sequencing whole genome libraries). For orthologous genes among *Quercus* and *Arabidopsis*, nucleotide diversity at synonymous sites (π_S_ = 0.004) within *Q. lobata* was also consistent with other trees [57] and plants in general [58]. Nucleotide diversity is an unbiased estimate of the population mutation rate (θ = 4*N*_e_μ) and thus independent of sample size [59, 60], although it too depends somewhat on the ability to identify SNPs and accurately genotype, which in turn can depend on sample size and coverage. SNPs in gene model coding regions also led to amino acid change distributions consistent with those observed in *Arabidopsis* (Additional files 12) and organisms generally (Additional file 13). Further, genotype calls were largely in line with expectations under Hardy-Weinberg equilibrium (Additional file 14). A number of factors can be attributed to the slight deviations seen from Hardy-Weinberg expectations, given the overly simplistic biological assumptions of that model. Notably, where the deviations are largest is where there is by far the least data, thus at most loci the genotype frequency is consistent with expectations. Overall, these comparisons suggest that the final list of called SNP loci and associated genotypes are generally of high quality.

### Patterns of molecular evolution among species

**Fig. 1:**
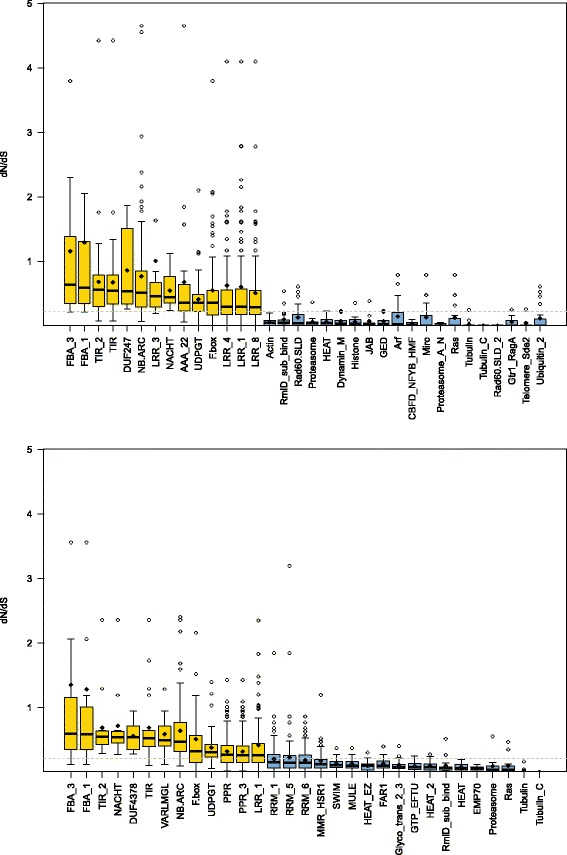
*d*_N_/*d*_S_ by gene functional category. Boxplots of functional gene categories for Pfam accessions associated with significantly high (yellow) or low (blue) *d*_N_/*d*_S_ for the comparisons of (**a**) *Quercus lobata* with *Q. garryana* and (**b**) the *Q. lobata*–*Q. douglasii* hybrid with *Q. garryana*. A few extreme *d*_N_/*d*_S_ > 5 are not shown. Overall medians are marked as gray dashed lines, and the means for each accession are shown as small black diamonds

Overall, *d*_N_/*d*_S_ ratios are well below unity (mean = 0.32 to 0.38, median = 0.21 to 0.22) (Additional files 15 and 16), suggesting a general influence of purifying selection in both oak species comparisons across most protein-coding regions, as has been observed in other plants [61]. The genes most under the influence of purifying selection (i.e., with *d*_N_/*d*_S_ near zero) in both comparisons included primarily structural or “housekeeping” genes, such as tubulin, actin, histones, ubiquitin, elongation factors, and ribosomal proteins (Fig. 1 and Additional files 16, 17, and 18). This is also consistent with the contigs of highest expression having slightly lower SNP locus rates than those of medium expression levels (Additional file 11).

In sharp contrast, disease response, abiotic stress response, regulatory, and growth and flowering genes have among the highest *d*_N_/*d*_S_ ratios. In the comparison of *Q. lobata* to the outgroup *Q. garryana*, genes containing 14 specific Pfam accessions had significantly higher *d*_N_/*d*_S_ at a threshold of *Q* < 0.01 compared to the background (all other genes) (Fig. 1a, Additional file 17), and in the comparison of the *Q. lobata–Q. douglasii* hybrid to *Q. garryana*, genes containing 13 accessions have significantly higher ratios than the background genes, and these accessions largely overlapped with the former 14 (Additional file 18). Combined, 11 of 18 distinct Pfam accessions associated with significantly high oak *d*_N_/*d*_S_ ratios are directly linked to plant disease resistance and immune response in crop or model system plants. These especially include the TIR (Toll/interleukin-1 receptor homology) domain, the NB-ARC signaling domain, the NACHT domain (related to NB-ARC), leucine-rich repeats (LRR), and F-box associated families [62–67]. The RPW8 family, which confers broad-spectrum mildew resistance in *Arabidopsis* [68], also had high *d*_N_/*d*_S_ (*Q* = 0.016). In all, at least 50 individual genes with evidence for involvement in disease resistance had *d*_N_/*d*_S_ > 1, of which 9 have GO associations to biotic stress response (Table 2) and 41 are members of gene families well-documented in the literature [62–66] (Additional file 16). High *d*_N_/*d*_S_ at disease resistance genes has also been observed in *Arabidopsis* and other plants [69–71].

**Table 2:** Abiotic or biotic stress response and flowering/seed development genes with *d*_N_/*d*_S_ > 1

Gene	Protein product	Gene symbol	Pfam or TAIR id(s)	*d*_N_/*d*_S_	
				*Q. lobata v. Q. garryana*	hybrid *v. Q. garryana*
***Biotic or abiotic response***					
m01oak04128cC-t01.1	Homeodomain-like superfamily protein	MEE3	AT2G21650	[10]	—
**m01oak00269cC-t01.1**	**light-harvesting complex of photosystem II 5**	**LHCB5**	**AT4G10340**	**6.66**	**4.60**
m01oak09138CC-t01.1	co-factor for nitrate, reductase and xanthine dehydrogenase 7	CNX7	AT4G10100	6.49	[10]
m01oak41018Ci-t01.1	TIR domain		PF13676; PF01582	4.42	—
m01oak10842cC-t01.1	growth-regulating factor 5	GRF5	AT3G13960	3.36	—
m01oak08493CC-t01.1	Drought-responsive family protein		AT4G02200	2.94	2.64
m01oak27235Ct-t01.1	MatE		PF01554	2.72	—
m01oak08705CC-t01.1	DNAJ heat shock N-terminal domain-containing protein		AT5G18750	2.36	0.32
m01oak14478Cc-t01.1	cell wall / vacuolar inhibitor of fructosidase 2	C/VIF2	AT5G64620	2.34	—
m01oak12092Cf-t01.1	Mlo family		PF03094	2.02	—
m01oak06031CC-t01.1	Mlo family		PF03094	1.83	—
m01oak08883CC-t01.1	hydroxyproline-rich glycoprotein family protein	ELF3	AT2G25930	1.78	1.38
m01oak35884CF-t01.1	TIR domain		PF13676; PF01582	1.76	—
m01oak20809ct-t01.1	DNA mismatch repair protein MutS, type 2		AT1G65070	1.76	—
m01oak11848cC-t01.1	Leucine-rich repeat (LRR) family protein		AT5G66330	1.62	—
m01oak08297cC-t01.1	ATP binding microtubule motor family protein		AT5G02370	1.61	1.52
m01oak01508Ct-t01.1	hydroxymethylbilane synthase	HEMC	AT5G08280	1.57	—
m01oak34476cC-t01.1	endonuclease V family protein		AT4G31150	1.56	—
m01oak17475CC-t01.1	5ʹ-3ʹ exonuclease family protein		AT1G01880	1.55	1.01
m01oak50068jm-t01.1	*Arabidopsis* broad-spectrum mildew resistance protein	RPW8	PF05659	1.43	—
m01oak16289cT-t01.1	chitin elicitor receptor kinase 1	CERK1	AT3G21630	1.38	—
m01oak01759cF-t01.1	TIR domain		PF01582	1.34	1.73
m01oak44069Cf-t01.1	TIR and NB-ARC domains		PF13676; PF01582; PF00931	1.28	0.30
m01oak10547CC-t01.1	abscisic acid responsive elements-binding factor 2	ABF2	AT1G45249	1.27	1.19
m01oak02511cC-t01.1	Auxin-responsive family protein		AT3G25290	1.23	[10]
m01oak14169cC-t01.1	Zinc finger C-x8-C-x5-C-x3-H type family protein	FES1	AT2G33835	1.20	0.60
**m01oak16383cC-t01.1**	**K** ^**+**^ **transporter 1**	**KT1**	**AT2G26650**	**1.15**	**0.68**
m01oak06110CC-t01.1	SUPPRESSOR OF AUXIN RESISTANCE 3	SAR3	AT1G80680	1.10	0.88
m01oak00652cC-t01.1	DUTP-PYROPHOSPHATASE-LIKE 1	DUT1	AT3G46940	1.10	1.02
m01oak00240cC-t01.1	germin 3	GER3	AT5G20630	1.10	—
**m01oak02432Ct-t01.1**	**Late embryogenesis abundant (LEA) hydroxyproline-rich glycoprotein family**	**NDR1**	**AT3G20600**	**1.02**	**1.09**
m01oak06210CF-t01.1	photolyase 1	PHR1	AT1G12370	1.02	1.60
m01oak06777CC-t01.1	F-box family protein		AT2G16365	0.75	1.08
m01oak09461cT-t01.1	cyclic nucleotide-binding transporter 1	CNBT1	AT3G17700	0.53	1.04
m01oak10758Ct-t01.1	ARP protein (REF)	NQR	AT1G49670	0.52	1.09
m01oak09684cC-t01.1	SBP domain		PF03110	0.51	2.46
m01oak11993SC-t01.1	purine permease 10	PUP10	AT4G18210	0.46	1.70
m01oak08222Cf-t01.1	COP1-interacting protein 7	CIP7	AT4G27430	0.45	1.37
m01oak14212jC-t01.1	HhH-GPD base excision DNA repair family protein		AT4G12740	0.41	1.21
m01oak18107cC-t01.1	5ʹ-3ʹ exonuclease family protein		AT1G18090	0.23	1.15
m01oak25703cC-t01.1	UDP-glycosyltransferase 73B4	UGT73B4	AT2G15490	0.01	1.39
***Flowering and seed development***					
m01oak10875CT-t01.1	Male sterility protein; 3-β hydroxysteroid dehydrogenase/isomerase, NAD dependent epimerase/dehydratase families		PF07993; PF01073; PF01370	6.26	—
m01oak08883CC-t01.1	hydroxyproline-rich glycoprotein family protein	ELF3	AT2G25930	1.78	1.38
m01oak26837JF-t01.1	S-locus glycoprotein family; D-mannose binding lectin; PAN-like, protein kinase, protein tyrosine kinase domains		PF08276; PF00954; PF01453; PF00069; PF07714	1.69	0.85
m01oak01757cC-t01.1	Glucose-methanol-choline (GMC) oxidoreductase family protein	HTH	AT1G72970	1.53	1.26
m01oak12787cC-t01.1	myosin heavy chain-related; maternal effect embryo arrest 13	MEE13	AT2G14680	1.30	0.47
m01oak04795cc-t01.1	Enoyl-CoA hydratase/isomerase family	AIM1	AT4G29010	1.25	0.80
m01oak28949ci-t01.1	S-locus glycoprotein family; D-mannose binding lectin		PF00954; PF01453	1.16	—
m01oak19887cC-t01.1	S-locus glycoprotein family; D-mannose binding lectin; Protein kinase, protein tyrosine kinase domains		PF00954; PF01453; PF00069; PF07714	1.02	—
m01oak09684cC-t01.1	SBP domain		PF03110	0.51	2.46
m01oak11825cC-t01.1	TCP-1/cpn60 chaperonin family protein; embryo defective 3007	EMB3007	AT5G18820	0.33	1.66

Forty-one other genes with *d*_N_/*d*_S_ > 1 that are involved in disease response inferred by Pfam hits such as NB-ARC, LRR, NACHT, and FBA are not shown because their Gene Ontology annotations did not explicitly link them to disease despite strong evidence in the literature. The three genes with Fay and Wu’s *H* < −2 are in bold

Within species, high genetic diversity at disease resistance genes is thought to play a role in conferring resistance to a broad diversity of pathogens and has been widely observed in plants [66, 72, 73] and animals [74]. For example, pathogen pressure on common alleles can lead to positive selection for rare alleles, thus promoting diversity [69, 75]. When biotic conditions change among populations or lineages, this underlying mechanism can continue, leading to diversifying selection among species for different alleles or suites of alleles. Thus, our observation that disease response genes are under divergent selection is consistent with the general observation that diseases, parasites (e.g., gall wasps and mistletoes), and fungal associates (e.g., mycorrhizal fungi) are often specific to particular plant species, including oaks [76–78]. Such pathogen pressure has been implicated as an important factor explaining biodiversity in tropical forests [79, 80].

The remaining Pfam accessions associated with significantly high *d*_N_/*d*_S_ are involved in a variety of functions. UDPGT-containing genes are involved in pigment biosynthesis in plants as well as toxin removal in mammals. PPR (pentatricopeptide repeat) genes are involved in organelle biogenesis and bind chloroplast RNA, suggesting a potential role in RNA editing [81, 82]. AAA (ATPase) genes are involved in a variety of functions, especially signal transduction and gene expression regulation. F-box associated (FBA) genes perform diverse roles in plants, including flowering and growth regulation, self-incompatibility, leaf senescence, and various abiotic and biotic responses [67]. In addition, the S-locus glycoprotein (S_locus_glycop) family related to self-incompatibility had significantly high *d*_N_/*d*_S_ (*Q* = 0.02), and three specific genes containing copies of it had *d*_N_/*d*_S_ > 1 (Table 2). Although variation at S-loci is often found to predate speciation [83, 84], the role of these loci in mating compatibility and reproduction gives them potential to be involved in divergence in some contexts. Several other genes influencing floral and embryo development also had *d*_N_/*d*_S_ > 1, consistent with the idea that some reproductive elements might differ among species. Finally, a number of abiotic response genes had high *d*_N_/*d*_S_, including those involved in light response (e.g., COP1-interacting protein 7, light-harvesting complex of photosystem II 5, and photolyase 1), heat shock (DNAJ heat shock N-terminal domain-containing protein), and drought (drought-responsive family protein) (Table 2).

**Fig. 2:**
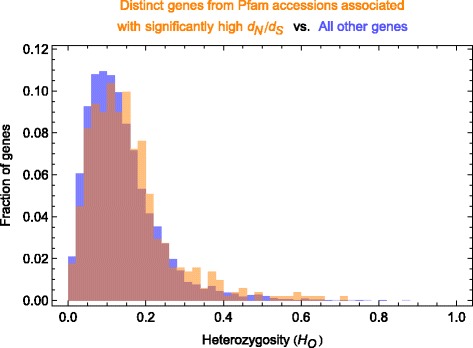
Mean observed heterozygosity per gene. Histograms of mean observed heterozygosity (*H*_O_) for each gene containing Pfam accessions with significantly high *d*_N_/*d*_S_ (orange) versus all other genes (blue)

To consider the possibility that high *d*_N_/*d*_S_ ratios might be attributed to variant calling on paralogous gene copies, we examined observed heterozygosity (*H*_O_) in high-*d*_N_/*d*_S_ genes versus all other genes. Paralogous gene copies are expected to diverge functionally and thus diverge at nonsynonymous sites [85], representing a potential problem if we inadvertently calculated *d*_N_/*d*_S_ for paralogs that were incorrectly collapsed to fewer copies during assembly. Collapsed paralogs are expected to show high levels of apparent heterozygosity (near 1.0) because the “SNPs” would, in fact, largely be fixed differences among the gene copies present in every sample. Reassuringly, we observe only a few *H*_O_ > 0.8 and no substantive difference between *H*_O_ in high-*d*_N_/*d*_S_ genes and most other genes (Fig. 2), indicating that few if any genes represent collapsed paralogs. Thus, the signal of divergent selection that we observe is likely real for the identified Pfam accessions and for most genes.

As complementary tests of positive selection in *Q. lobata* relative to its ancestor with *Q. garryana*, we calculated for each gene Fay and Wu’s *H*, which is expected to have negative values under positive selection [86]. We did not find any class of genes defined by the Pfam accessions they contained to have unusually negative or positive *H* (*Q* > 0.39) (Additional file 19). However, 1,380 genes have low values (*H* < −2), and 151 individual genes have very low values (*H* < −5), suggestive of positive selection (Additional files 15 and 20). Among all these, 37 also had *d*_N_/*d*_S_ > 1, of which 4 likely play a role in abiotic or biotic stress response: light-harvesting complex of photosystem II 5 (m01oak00269cC-t01.1; *H* = −3.38), K^+^ transporter (m01oak16383cC-t01.1; *H* = −2.73), late embryogenesis abundant (LEA) hydroxyproline-rich glycoprotein family (m01oak02432Ct-t01.1; *H* = −2.92), and an AAA domain-containing protein (m01oak15073cF-t01.1; *H* = −4.01) (Table 2). The strongest evidence for positive selection is for light-harvesting complex of photosystem II 5, which has both very high *d*_N_/*d*_S_ (Table 2) and low *H*. However, there was no overall correlation of *d*_N_/*d*_S_ with *H* (*r* = 0.03). Given our limited sampling, our primary goal was to identify functional classes of genes under positive selection, and it appears that the signal in *H* is not strong enough to independently confirm the *d*_N_/*d*_S_ results.

In summary, we found preliminary support for divergent selection on biotic and abiotic stress response genes based on *d*_N_/*d*_S_, consistent with the hypothesis that ecological forces are important in the divergence and potentially in the maintenance of species boundaries in California oaks. Identifying the classes of genes under divergent selection provides insight into the factors most strongly involved in divergence among these hybridizing taxa, but warrants future direct investigation of the role of ecological factors in speciation and maintenance of species boundaries.

## Conclusions

We have constructed a draft transcriptome for several California oaks that includes transcript contig nucleotide sequences, gene models (including CDS, UTR, and intron annotations), and functional associations based on Pfam domain occurrences and gene orthologs with *Arabidopsis*. Although understandably not at the quality of a model organism, this transcriptome appears to be a large representative fraction of transcribed loci in *Quercus* and is the most complete for this genus reported to date. Many gene models are of good quality and complete, many transcripts correspond to exactly one gene, and individual genes are generally not multiply assembled, even though *Quercus* has relatively high genetic variation across individuals. We further identified over 1.1 million high-quality diallelic SNP loci and genotyped 24 individuals, representing the largest SNP resource available in *Quercus* [5] and among the highest for any tree [51–53]. This transcriptome provides a solid foundation for studies of genetic variation and gene expression in *Quercus*, and will enable research in comparative genomics, phylogeography, hybridization, adaptation genomics, and quantitative genetics in oaks.

By investigating patterns of nonsynonymous and synonymous SNP variation among classes of genes among species, we found preliminary support that biotic stress is a major factor in divergent evolution among oak species. By implication, selection for stress response could contribute to the maintenance of species boundaries among these hybridizing species. Future work that includes more species and codon-level analyses, as well as studies of genomic patterns of introgression along hybrid zones, will shed more light on the specific loci that are most important in speciation, species integrity, and adaptation.

## Methods

### Sampling and sequencing

The first week of April 2011, we sampled bud, expanding leaf/flower bud, young leaf and/or young male flower tissue from 22 *Quercus lobata* individuals spread throughout its entire distribution, 1 *Q. garryana* individual, and 1 probable *Q. lobata*–*Q. douglasii* hybrid individual (Table 1). The *Q. garryana* was sampled to permit interspecific comparison, while *Q. lobata* was more intensively sampled to generate a SNP resource for other studies. The hybrid was originally collected under the assumption that it was *Q. lobata* because of its relatively deeply lobed leaves, but it was later determined that it had intermediate leaf characteristics consistent with other hybrid *Q. lobata* × *Q. douglasii* (Gugger PF, unpublished data) [14]. In addition, this sample is identified as extraordinarily different from both *Q. lobata* and *Q. garryana* in a principal components analysis on SNPs, and across SNPs it is heterozygous unusually often, suggesting recent hybrid origin (Additional file 21). *Q. douglasii* and *Q. lobata* co-occur at the collection site. Samples were immediately frozen on dry ice in the field and stored at −80 °C until total RNA extraction. Preliminary RNA precipitation (http://openwetware.org/wiki/Conifer_RNA_prep) was followed by the Qiagen RNeasy Plant Mini Kit with DNase treatment protocol. RNA-Seq libraries with insert lengths 100–380 bp (mode = 170 bp) as determined by BioAnalyzer (Agilent) were prepared from 4 μg of RNA using an Illumina TruSeq RNA Sample Prep Kit. Each library was uniquely tagged using Illumina TruSeq indexed adapters #1 to #12 to enable equimolar 12-plexing of samples, using two lanes to accommodate the 24 individuals. Sequencing was paired end 100 + 100 bases on Illumina HiSeq 2000 v3 flow cells at the Neuroscience Genomics Core, UCLA.

### Transcriptome assembly and quality

Only read pairs with Illumina RTA PF = 1 and perfect matches to an expected 6-mer index tag were retained. Adapters were investigated using lists of known Illumina adapter and library preparation-related sequences (allowing substitutions and indels, as well as fragmentary matches anywhere within reads and these sequences) as well as *de novo* assemblies of over-represented *k*-mers from read tails. Paired read ends that unambiguously overlapped by ≥ 16 bases with ≤ 3 mismatches were merged to produce a large population of longer virtual single end reads, taking advantage of double sequencing in the overlap to lower basecall errors. Read pairs not overlapping were retained as ordinary paired ends. Each read was cut to the longest interval with ≤ 1 N that does not start or end with an N (resolving any ties in favor of the 5ʹ-most interval); if < 50 bases remained, the read was discarded, and otherwise any Ns were replaced by independent uniformly random choices from A/C/G/T. Surviving single and paired end sequences were given to Ray 2.2.0 (*k* = 51) for the primary *de novo* assembly step [35]. As already described, subsequently some contigs were merged, and then some were split. The resulting contigs contain no IUPAC ambiguous nucleotides or gaps.

Evidence for quality of the transcriptome has already been presented earlier; only methodological details missing in those discussions are given here in Methods. For testing the fraction of original reads mapping to the transcriptome assembly, Bowtie 2.1.0 [87] was used set to ‘--sensitive-local’. Identification of rRNA contigs to investigate contamination was as follows (although not poly-adenylated, rRNA is often sufficiently abundant in total RNA that it survives in RNA-Seq experiments prepared with poly-A purification). From a 39,412-sequence multiple alignment of large-subunit rRNA across the entire tree of life from SILVA 115 [88], a consensus of a 1 kbp “core” interval of high conservation was identified and used as a BLASTn 2.2.26 (E-value threshold 10^−6^) target for both strands of transcriptome contigs. The entire, end-to-end sequences of contigs with alignments were given to NCBI web BLASTn to the ‘nr’ database to identify high-coverage, high-identity hits to known sequences, with the entire, end-to-end known sequences then used as new targets to align original read pairs against with Bowtie2 [87].

The EST-based transcript data for other *Quercus* species are *Q. alba* from eastern North America (WO454 Unigene V2, which includes aboveground and belowground tissues) [9] and *Q. robur*/*Q. petraea* from Europe (OakContigV1, which includes leaves, buds, flowers, pollen) [5]. Using USEARCH 7.0 [89] (with thresholds of 92 % nucleotide identity and E-value 10^−10^; conclusions are insensitive to reasonable modifications), we estimated reciprocal overlap in the number of transcripts among our data and the other oak species.

### Repeats and transposons

Tandem Repeats Finder 4.04 [36] parameters were 2 7 7 80 10 50 2000. RepeatMasker 3.3.0 (http://www.repeatmasker.org/) with Repbase 2011–09–20 (using all of Eukaryota) [90] was run on the final transcriptome, with identified repeats presented in lowercase in the nucleotide sequences of deposited contigs.

### Gene models

Bootstrapping of gene models began with an *ab initio* run of GlimmerHMM 3.0.2 [45] with stock *Arabidopsis* parameters [91]. The subset of models containing introns were further filtered before training: only models with 1–3 introns were allowed as candidates, and these were aligned as amino acids with lastal (−m 100 -j 3) of LAST 189 [92] to a large collection (45 M sequences, 7 G amino acids) of NCBI reference proteins (‘nr’ 2011–11–30 04:12 + ‘env_nr’ 2011–11–19 22:13 + ‘pataa’ 2011–11–30 12:35), and only models with at least one alignment covering ≥ 95 % of the model and ≥ 95 % of the NCBI sequence were retained. Throughout this project, all amino acid translations were taken via the standard universal genetic code, NCBI #1. AUGUSTUS 2.5.5 [46] was then trained (on both the intronless and high quality intron models) and used iteratively as already described. Note that, even when calling genes on both strands, AUGUSTUS is surprisingly sensitive to the strand (Watson or Crick) presented to it; thus AUGUSTUS runs involved running AUGUSTUS twice, once for bi-strand calling on Watson and once for bi-strand calling on Crick strand, and an overlap resolution/non-redundant extraction procedure preferring complete models used to merge the results. The coding sequence of non-complete models may start and/or end on other than a codon boundary; such cases are properly represented, e.g., via the frame/phase (column 8) information in the deposited GFF/GTF file that communicates the models.

UTRs were only assigned to AUGUSTUS (CDS + intron) models that had both a start and stop codon, and additionally were the only model on their parent contig. If there were any nucleotides outside the span of the AUGUSTUS model and upstream of it on its strand, they were all taken as 5ʹ-UTR in a single interval. Similarly, if there were any nucleotides outside the model and downstream of it on its strand, they were all taken as 3ʹ-UTR in a single interval. No UTRs were assigned to models on contigs with multiple models, or to models lacking either a start or stop codon. No attempt was made to identify any introns in UTRs.

### Functional annotation

The process of determining draft orthologs with *Arabidopsis* began with BLASTp 2.2.26 (E-value threshold 10^−6^) amino acid alignments in both directions between all 35,386 TAIR10 models (splice variants included) and the final AUGUSTUS *Quercus* proteins (both complete and partial, with final STOP removed when present and each internal STOP [rare] replaced with an x). For each direction, for each query, only hits with bitscore ≥ 99 % of the top bitscore for that query were retained; then, for each subject, only hits with bitscore ≥ 99 % of the top bitscore for that subject were retained. Form a bipartite digraph with vertices the TAIR10 genes (dropping splice variant distinctions) and oak models, with arcs from queries to subjects, and collapse parallel multiple arcs to single arcs. Remove all vertices except those with exactly one outgoing arc, and declare as putative orthologous pairs those pairs of vertices with arcs pointing reciprocally at each other. These orthologs provide inferred gene annotation information for oak in the form of protein product names and associated GO (accessed 2014-03-25) controlled vocabulary terms via TAIRs extensive curation of the *Arabidopsis* model organism [38, 49].

Annotational coverage of a larger fraction of oak models (at the expense of generally less specific information) is obtained by finding occurrences of Pfam accessions (domains, etc.) across all the gene models. HMMer 3.1b1’s [93] hmmsearch (leveraging Pfam’s carefully chosen high specificity, high sensitivity per-accession thresholds with the --cut_tc option) was used to identify occurrences of Pfam 27.0 A [33, 34] accessions via their consensus HMM amino acid profiles. As Pfam to Gene Ontology associations are available (http://geneontology.org/external2go/pfam2go, downloaded 2014-06-23), these provide additional inferred GO associations for oak models.

In each case, the inferential (restricted transitive) closure of Gene Ontology associations was taken, using the conventional inference rules recommended by GO.

### Variant calling

Variants were called using a pipeline based on GATK 2.8.1 (2.5.2 for steps before genotyping) [50], roughly following GATK best practices [94, 95]. GATK is, however, primarily designed for low coverage genomic re-sequencing of mammalian model organisms. Adaptations and parameter choices are needed to apply it to RNA-Seq reads (with their highly variable coverage reaching extraordinary levels for the highest genes) and *de novo* assembled contigs in a non-model organism such as oak, not only for computational considerations but also quality of output. The pipeline begins with the final draft oak transcriptome contigs as reference and the Illumina RTA PF = 1 paired-end 100 + 100 base reads tagged by individual with per-base RTA Phred-scale quality scores (with Illumina EAMSS quality score adjustments applied) as reads.

Reads were aligned to the reference with Bowtie 2.1.0 [87] as paired ends (−q --very-sensitive-local --n-ceil L,2,0 --dpad 12 --gbar 5 --score-min L,102,0 --minins 75 --maxins 500 --fr --no-dovetail --no-contain) to produce per-individual SAM files that were converted to BAM and sorted by aligned position on the reference. Alignments were filtered to only retain those with MAPQ ≥ 10 and FLAG bitwise AND with 0xF04 being zero (segment not unmapped, and not secondary alignment, and flagged as passing quality controls, and not flagged PCR/optical duplicate, and not a supplementary alignment). For each position on each reference contig, for each individual, if there were multiple reads whose alignments started at the position, then only a single one selected uniformly at random was retained; this capped coverage to a high but computationally manageable level for GATK, while maintaining high diversity of contributing reads. The resulting BAM files were merged into a single BAM (maintaining tags marking individuals).

GATK RealignerTargetCreator (−−downsampling_type NONE --baq OFF --windowSize 10 --mismatchFraction 0.0 --minReadsAtLocus 4 --maxIntervalSize 500) and then IndelRealigner (−−downsampling_type NONE -- baq CALCULATE_AS_NECESSARY --baqGapOpenPenalty 30 --LODThresholdForCleaning 5.0 --consensusDeterminationModel USE_READS --entropyThreshold 0.15 --maxReadsInMemory 150000 --maxIsizeForMovement 3000 --maxPositionalMoveAllowed 200 --maxConsensuses 30 --maxReadsForConsensuses 240 --maxReadsForRealignment 20000) were run (partitioning contigs into 84 piles with approximately equal numbers of aligned reads and running piles in parallel). As no high quality, near complete file of already known variants was available, GATK base score quality recalibration (BSQR) was skipped.

Numerous experimental trial runs of GATK HaplotypeCaller and UnifiedGenotyper were made, evaluating outputs by various statistical measures as well as by detailed hand examination (using the IGV genome browser [96]) of variant/genotype calls and aligned reads for a small random contig sampling. Considerations of output quality and total computational effort required led to a decision to determine possible alleles at each reference position outside of GATK and then use GATK UnifiedGenotyper with certain parameters (see below) to genotype individuals against these possible alleles. Formation of possible alleles began with the Bowtie2 original SAM outputs pooled across individuals, filtering to retain alignments with MAPQ ≥ 20 and FLAG bitwise AND with 0x704 being zero. Each alignment is broken into maximal runs of four types: insert-to-reference, deletes-from-reference, basepairs-in-1:1-correspondence-and-different, and basepairs-in-1:1-correspondence-and-identical; runs of the last type were dropped. The number of times observed for each distinct quintuple of run type, contig name, position on contig (which is between two reference basepairs for inserts to reference), reference sequence (for + strand), and read sequence (as if + strand) was determined, and only quintuples seen ≥ 5 times (and with read sequence having no IUPAC ambiguous nucleotides) were retained; those that survive constitute the tentative possible alleles. The tentative alleles were converted to VCF file format (by grouping alleles at each position into a locus, and, since VCF requires all alleles to be non-empty, loci that would otherwise involve an allele being a string of zero nucleotides [e.g., indels] were grown to include one more upstream nucleotide on the + strand, in the usual way for VCF). As GATK UnifiedGenotyper does not fully handle multiple VCF loci overlapping on the reference, each chain of overlapping tentative loci was merged (with a custom script, as GATK CombineVariants was found insufficient) into a single variant locus (growing the extent involved on the reference and adjusting alleles appropriately in response). The resulting VCF file defines the possible alleles that were used as the genotyping targets in the next paragraph.

Genotyping against the possible alleles was conducted with GATK UnifiedGenotyper (−−downsampling_type NONE --baq CALCULATE_AS_NECESSARY --baqGapOpenPenalty 30 --defaultBaseQualities 30 --heterozygosity 0.05 --indel_heterozygosity 0.005 --genotyping_mode GENOTYPE_GIVEN_ALLELES --input_prior 0.020408163265306 «…48 copies total; 0.0204… is 1/49» --input_prior 0.020408163265306 --standard_min_confidence_threshold_for_calling 10 --standard_min_confidence_threshold_for_emitting 10 --max_alternate_alleles 24 --contamination_fraction_to_filter 0.0 --pcr_error_rate 0.0001 --computeSLOD --annotateNDA --pair_hmm_implementation LOGLESS_CACHING --min_base_quality_score 20 --max_deletion_fraction 9.99 --allSitePLs --min_indel_count_for_genotyping 5 --min_indel_fraction_per_sample 0.25 --indelGapContinuationPenalty 10 --indelGapOpenPenalty 30 --sample_ploidy 2 --output_mode EMIT_VARIANTS_ONLY), once (the “SNV run”) specifically for single-nucleotide variants (−−genotype_likelihoods_model SNP, where reference and all alternate alleles are single nucleotides) and once (the “MNV” run) to add multi-nucleotide and indel variants (−−genotype_likelihoods_model BOTH, with reference and/or at least one alternate allele being other than a single nucleotide). Runs were merged with GATK CombineVariants (−−downsampling_type NONE --baq OFF --genotypemergeoption PRIORITIZE --rod_priority_list SNV,MNV --filteredrecordsmergetype KEEP_IF_ANY_UNFILTERED --printComplexMerges --excludeNonVariants --minimumN 1 --combineAnnotations). These jobs were broken into many parts run in parallel with individual parts run with GATK (−nt/-nct) threading; crashing of some parts was alleviated by re-running them without GATK threading. About 200 loci with 25+ possible alleles could not be fully processed. GATK variant quality score recalibration (VQSR) was skipped as no pre-existing high quality file of variants existed. The output VCF file is the “master” (unfiltered) deposited set of variants (with a reference and one or more variant [non-reference] alleles specified at each locus), genotypes (with each individual at each locus either assigned to two [not necessarily distinct] alleles, or left uncalled and assigned to no alleles), and a large variety of embedded statistics.

Our focus in this work is on diallelic SNPs. By examination of the distributions of the many metrics that GATK includes in its output VCF files and how statistical properties of variants (e.g., transition:transversion ratio) behave in different regimes of these metrics, filtering criteria for SNVs were established. Downstream analyses were restricted to SNP loci that satisfied all of the following: 17.0 ≤ QUAL < 100000.0, total AC is not 0 or 48, −3.5 < BaseQRankSum < 7.0, DP ≥ 5, Dels < 0.1, FS < 90.0, HaplotypeScore < 25.0, MQ ≥ 20.0, −25.0 < MQRankSum < 10.0, −3.5 < ReadPosRankSum < 9.0, and SB < 3.0; and further, individual genotype calls were filtered as follows (changing to uncalled those that fail either condition): DP ≤ 100 and GQ ≥ 20. Experimentation with GQ thresholds of 0, 10, 27, and 44 was also conducted. Downstream diallelic SNP analyses were performed on those surviving loci that had exactly two alleles represented across surviving genotypes, with one of these being the reference allele (and both being single nucleotides); these are the loci referred to in this work as “SNPs” unless otherwise specified.

Filtered diallelic SNP loci were divided into groups based on their position on the reference relative to gene models (i.e., start codons, stop codons, interior codons, introns, 5ʹ-UTR, 3ʹ-UTR, and remaining base pairs). The predicted coding effect of each variant allele landing in a codon was made by a script that considered each locus in isolation and examined the reference codon versus the alternate codon induced by the variant allele as interpreted by the standard universal genetic code, and codons with multiple loci were generally excluded from downstream analyses. A VCF-to-VCF run (in 100 parts) of SnpEff 3.2a [97] (with the oak transcriptome and gene models added) was also performed (−lof -oicr), although it was found that (although some of its source code seems to exist for the purpose) models not starting/ending on a codon boundary generated errors/warnings so that models not starting on a codon boundary had to be suppressed from the oak reference presented to it and its output does not contain all effects for all models.

For the Hardy-Weinberg analysis, allele frequencies were parameterized as the two-dimensional space of real triples (*r*, *h*, *v*) with *r*, *h*, *v* ≥ 0, *r* + *h* + *v* = 1, and *r*, *h*, and *v* defined as the fractions of individuals that are homozygous reference, heterozygous, or homozygous variant, respectively. Hardy-Weinberg equilibrium is a particular one-dimensional curve in this space. To estimate an empirical observed density from the oak data, only the 76,233 filtered diallelic SNP loci that, on restriction to the 22 *Q. lobata* individuals, had ≥ 11 called genotypes, ≥ 1 individual called homozygous reference, ≥ 1 individual called homozygous variant, and ≥ 1 individual called heterozygous were used. The empirical density was taken as the average over these loci of the posterior density from the observed *Q. lobata* genotype calls for that locus starting from a uniform Dirichlet prior, and the figure in Additional file 14 presents the logarithm of the result in equilateral barycentric coordinates (known in this context as a de Finetti diagram). Note that we do not expect (and do not observe) uniform distribution on the one-dimensional Hardy-Weinberg equilibrium curve; there is great enrichment for *r* to be high (as many alleles determined in this study occur in few individuals). Hence, we examined where the empirical density attains its maximum along each line from *h* = 1 to *h* = 0 (each such line crosses the Hardy-Weinberg equilibrium curve exactly once) and find this location to be near the Hardy-Weinberg equilibrium curve for all lines.

### Molecular evolutionary tests of divergent selection

We estimated the nonsynonymous versus synonymous substitution rates among species (*d*_N_/*d*_S_ = *K*_a_/*K*_s_) for the coding sequence of each gene model to identify genes and functional groups of genes under the influence of positive divergent (*d*_N_/*d*_S_ > 1) or purifying (*d*_N_/*d*_S_ < 1) natural selection among species lineages [26]. The *d*_N_/*d*_S_ rates were calculated from the filtered diallelic SNPs with C++ code equivalent to dnds(…, ‘METHOD’,‘PBL’, ‘WINDOW’,inf, ‘ADJUSTSTOPS’,true) revision 1.1.8.13 from MATLAB Bioinformatics Toolbox 4.0 (MathWorks, Inc., Natick, MA, USA), which uses the Pamilo-Bianchi-Li method [98, 99] based on the Kimura two-parameter model [100] that keeps track of three levels of codon degeneracy and corrects for transition/transversion imbalance. We used this model to estimate *d*_N_, *d*_S_, and their variances by directly comparing each individual to the outgroup *Q. garryana* [12]. For each gene, for each individual, two coding sequences (“haplotypes”) were formed. Each sequence has all coding nucleotides (variant or not) from the gene model, padded with ambiguous nucleotides to the nearest codon boundary on each end. Nucleotides not at a filtered diallelic SNPs are from the transcriptome contig (and common to all individuals), while at a filtered diallelic SNPs, the two sequences together represent the called genotype for the individual at that locus (and note that lack of haplotype phasing across such positions does not matter for dnds()’s calculations). Uncalled genotypes are replaced with ambiguous nucleotides. When two individuals are compared, haplotypes are concatenated, so that each coding position in the model contributes four times, once for each pairing of haplotype in one individual to haplotype in the other individual. We also tried alternative methods of estimating *d*_N_/*d*_S_ by inferring an “ancestral” sequence for comparison to each extant individual’s sequence [101], as well as a variety of other simpler methods [102, 103], but the results were highly similar, and thus only the more conventional approach described first is presented here.

The bulk of *d*_N_/*d*_S_ values were found to generally follow log-normal distributions (e.g., normal distributions after log_2_-transformation). Hence, when summarizing the 22 *Q. lobata d*_N_/*d*_S_ values for a gene or class into a single value, a geometric mean was taken. To identify unusually extreme summarized gene values on log_2_ scale, values outside [−5.0, 1.0] were temporarily ignored (removing tails completely) and parameters of a normal distribution truncated to this interval determined by maximum likelihood. Tail probabilities of the untruncated log-normal (after going back to full range on linear scale) then provide a measure of unusualness; the one-tail α = 0.05 levels for high *d*_N_/*d*_S_ values are those ratios above 0.99 for the *Q. lobata* versus *Q. garryana* comparison and above 0.85 for the *Q. lobata*–*Q. douglasii* hybrid versus *Q. garryana* comparison. Hence, *d*_N_/*d*_S_ > 1 not only represents potentially divergent selection, but is also significantly higher than the bulk of ratios in both comparisons.

When closely related species are compared, some SNPs might still be segregating and thus not fixed among species. This fact has the potential to render *d*_N_/*d*_S_ less sensitive to selection intensity making it a conservative test for positive selection, especially in the most extreme case of a single population [104, 105]. However, our data set represents a more favorable scenario of substantial divergence despite some shared polymorphism, suggesting that *d*_N_/*d*_S_ is appropriate but underpowered for identifying cases of divergent selection [104]. *d*_N_/*d*_S_ can also be considered a conservative test for positive selection when estimated along entire coding regions as we do because different sites within genes might be under different selection pressures [26]. In the case of low *d*_N_/*d*_S_, intraspecific and interspecific estimates are highly correlated in practice, regardless of theoretical considerations [106]. Nonetheless, we cautiously used *d*_N_/*d*_S_ primarily for assessing differences among broad functional categories of genes as defined by those that contain specific Pfam accessions, rather than making strong claims about specific loci. Functional classes of genes based on Pfam accessions were tested for unusually high or low values of *d*_N_/*d*_S_ with Wilcoxon rank-sum tests [107, 108], and *P*-values were adjusted for multiple testing to *Q*-values using the false discovery rate method of Benjamini and Hochberg [109, 110]. Only rates computed from at least six substitutions of either kind (synonymous or not) were considered. If *d*_N_ was estimated as zero, we assigned *d*_N_/*d*_S_ = 0; if *d*_S_ was estimated as zero, we assigned *d*_N_/*d*_S_ = 10. Because of the use of rank-based statistical tests, results are not sensitive to reasonable alternative assignments in these cases.

To complement the *d*_N_/*d*_S_ tests, for each gene-containing contig we computed Fay and Wu’s *H* as an independent test for positive selection in *Q. lobata* relative to its ancestor with *Q. garryana. H* is expected to be negative when there is positive selection leading to high frequency of a derived allele. To calculate *H*, we used C++ code equivalent to faywu00h_test in the MATLAB PGEToolbox [111], and as the ancestral sequence, we used the *Q. garryana* allele, or, when missing, the most common allele across the *Q. lobata* individuals. The latter choice should bias the *H* estimates towards more positive values because the putatively derived allele would then be at lower frequency in *Q. lobata*, thus making the test more conservative for inferring positive selection. We allowed up to 4 of 44 missing alleles (*n* = 22) within *Q. lobata* at each site by randomly resampling 40 alleles at each SNP locus. *H* was calculated for ten such replicates and summarized with the median. Functional classes of genes were tested for unusually high or low *H* using Wilcoxon rank-sum tests, and the resulting *P*-values adjusted for multiple testing in the same way as for *d*_N_/*d*_S_.

Mean heterozygosity per gene was calculated for each gene with at least 6 SNPs genotyped in at least 10 of 22 *Q. lobata* individuals and the *Q. garryana* individual, ignoring the hybrid individual.

## Availability of supporting data

Illumina sequence reads, the final draft transcriptome contigs, annotations, variant calls, per-individual genotypes, and results of tests for natural selection are publically available through NCBI under project accession PRJNA282155 and/or a dedicated oak transcriptome assembly project page on the UCLA genomics resource website at http://genomes.mcdb.ucla.edu/OakTSA/.

## Additional files

Additional file 1:
**Transcriptome size and coverage per contig.** **(a)** Total transcriptome size in base pairs (red) and number of contigs (blue) as minimum contig size threshold in base pairs (*x* axis) is varied; and **(b)** histogram of log_10_ average coverage for all contigs (purple) versus contigs ≥ 1 kbp (green). (PDF 554 kb) Additional file 2:
**Uniqueness of contigs.** Distribution of transcriptome contigs by percent of 100-mers that are ≥ 10 mismatches away from the nearest other 100-mer in the entire transcriptome. (PDF 360 kb) Additional file 3:
**C+G content in oak and** ***Arabidopsis*** **.** Distribution of C+G content for certain populations of nucleotide sequences (oak solid lines, *Arabidopsis* dotted lines): coding sequences from all gene models (green, TAIR file TAIR10_cds_20110103_representative_gene_model_updated), 5ʹ-UTRs (blue, TAIR file TAIR10_5_utr_20101028), 3ʹ-UTRs (magenta, TAIR file TAIR10_3_utr_20101028), and entire contigs for oak contigs having no gene models (solid red) or all *Arabidopsis* intron sequences (dotted red, TAIR file TAIR10_intron_20101028). (PDF 392 kb) Additional file 4:
**Overlap in gene content among oak RNA data sets.** Pairwise comparison of percentage of transcripts from each transcriptome appearing in the other. (PDF 64 kb) Additional file 5:
**Comparison of sizes among oak RNA data sets.** Comparison of the *Quercus lobata* transcriptome of this work (all contigs) with the EST-based transcriptomes of *Q. alba* and *Q. robur*. (PDF 60 kb) Additional file 6:
**Comparisons of oak and** ***Arabidopsis*** **amino acid usage and length of gene models and UTRs.** **(a)** Relative frequency of amino acid usage in *Arabidopsis* TAIR10 gene models compared to complete *Quercus* gene models ordered left to right from most to least common; and distributions of the length of **(b)** gene models in amino acids, **(c)** 5′-UTRs in nucleotides, and **(d)** 3′-UTRs in nucleotides for *Arabidopsis* TAIR10 (green) versus complete *Quercus* models (red). (PDF 763 kb) Additional file 7:
**Summary of alignments between oak and** ***Arabidopsis*** **orthologs.** Histograms of **(a)** the percent of protein length participating and **(b)** the percent amino acid identity of amino acid alignments for *Arabidopsis*–*Quercus* orthologous genes. (PDF 394 kb) Additional file 8:
**Comparison of number of and expression level of gene models with and without oak-** ***Arabidopsis*** **orthologous pairing.** Histograms by expression level of **(a)** the number of gene models that are complete and have a called *Arabidopsis* ortholog (green), partial with an ortholog (yellow), complete without an ortholog (orange), or partial without an ortholog (red); **(b)** the number of contigs by pooled expression for all contigs (red) versus those with orthologs (green); and **(c)** the number of *Arabidopsis* genes by expression levels in a generic *Arabidopsis* RNA-Seq experiment (NCBI SRX145413 [47], red) versus only those with a called oak ortholog (green). (PDF 559 kb) Additional file 9:
**Expression level in oak versus** ***Arabidopsis*** **orthologs.** Two-dimensional histogram of pooled oak expression from oak transcriptome contigs versus expression of the orthologous *Arabidopsis* gene in a generic *Arabidopsis* RNA-Seq experiment (NCBI SRX145413 [47]). (PDF 647 kb) Additional file 10:
**Distributions of Gene Ontology terms for oak-** ***Arabidopsis*** **orthologs versus all** ***Arabidopsis*** **genes.** Distributions of Gene Ontology Plant Slim functional terms for the *Arabidopsis* side of the 9,431 *Quercus*–*Arabidopsis* ortholog gene pairs (outer rings) versus all *Arabidopsis* TAIR10 genes (inner ring) for **(a)** cellular components, **(b)** biological processes, and **(c)** molecular functions. (PDF 556 kb) Additional file 11:
**SNP locus rate in oak versus** ***Arabidopsis*** **.** Comparison of called SNP locus rate per base pair in **(a)** *Arabidopsis* and **(b)** oak by model-relative position averaged over gene models (stretching/compressing each transcription start to stop span to a nominal length of 2 kbp), including breakdown by coding (green), 5ʹ-UTR (blue), 3ʹ-UTR (light blue), intron (red), and all types combined (black, dashed); and **(c)** called SNP locus rate per oak base pair as a function of descending coverage, consistent with the low SNP locus rates at the edges of oak in (a) and (b) originating from low coverage at contig edges (and thus lower power to detect variants). (PDF 415 kb) Additional file 12:
**Pairwise amino acid changes inferred from SNPs inside coding sequence of gene models.** Amino acids are given by IUPAC single letter codes (with ‘*’ for STOPs). (PDF 75 kb) Additional file 13:
**Concordance of amino acid change distributions between oak and standard NCBI BLOSUM95.** (PDF 331 kb) Additional file 14:
**Consistency of called oak genotypes with Hardy-Weinberg equilibrium expectations.** Called oak genotypes within *Quercus lobata* achieve maximum density at black points based on the log posterior density (color gradient). Hardy-Weinberg equilibrium is the white curve. (PDF 351 kb) Additional file 15:
**Histograms of** ***d***_**N**_ **/** ***d***_**S**_ **ratios and Fay and Wu’s** ***H*** **.** **(a)** *d*_N_/*d*_S_ for *Quercus lobata* versus *Q. garryana* (red) and the *Q. lobata–Q. douglasii* hybrid versus *Q. garryana* (blue). **(b)** *H* for *Q. lobata* versus its inferred ancestor with *Q. garryana*. (PDF 225 kb) Additional file 16:
***d***_**N**_ **/** ***d***_**S**_ **for each sample at each gene containing at least six SNPs, along with associated Pfam and TAIR annotations.** (XLSX 7125 kb) Additional file 17:
**Summary statistics of** ***d***_**N**_ **/** ***d***_**S**_ **for** ***Quercus lobata*** **versus** ***Q. garryana*** **for genes containing each Pfam accession.** Results of Wilcoxon rank-sum tests for whether genes with each accession had higher or lower *d*_N_/*d*_S_ than typical genes are also given. (XLSX 115 kb) Additional file 18:
**Summary statistics of** ***d***_**N**_ **/** ***d***_**S**_ **for** ***Q. lobata–Q. douglasii*** **hybrid versus** ***Q. garryana*** **for genes containing each Pfam accession.** Results of Wilcoxon rank-sum tests for whether genes with each accession had higher or lower *d*_N_/*d*_S_ than typical genes are also given. (XLSX 106 kb) Additional file 19:
**Summary statistics of Fay and Wu’s** ***H*** **for** ***Q. lobata*** **versus inferred ancestor with** ***Q. garryana*** **for genes containing each Pfam accession.** Results of Wilcoxon rank-sum tests for whether genes with each accession had higher or lower *H* than typical genes are also given. (XLSX 165 kb) Additional file 20:
**Fay and Wu’s** ***H*** **among** ***Q. lobata*** **and its inferred ancestor with** ***Q. garryana*** **for each gene, along with associated Pfam and TAIR annotations.** (XLSX 2985 kb) Additional file 21:
**SNP evidence for hybrid.** **(a)** Principal components analysis of 226,423 SNPs from 24 *Quercus* samples, showing the 22 *Q. lobata* samples (clustered in the the lower right), the *Q. garryana* individual (in the lower left), and the probable *Q. lobata–Q. douglasii* hybrid individual (at the top); and **(b)** proportion of heterozygous genotype calls at SNPs for *Q. lobata* (blue), *Q. garryana* (green), and Springville 1 (red, the probable hybrid), with samples ordered left-to-right by latitude south-to-north. (PDF 364 kb)
