# Supplementary material for: Evolutionary insights from de novo transcriptome assembly and SNP discovery in California white oaks
Source: BMC Genomics. 2015 Jul 28;16(1):552. doi: 10.1186/s12864-015-1761-4 (PMC4517385; doi:10.1186/s12864-015-1761-4)
Supplement: Additional file 1: — Transcriptome size and coverage per contig. (a) Total transcriptome size in base pairs (red) and number of contigs (blue) as minimum contig size threshold in base pairs (x axis) is varied; and (b) histogram of log10 average coverage for all contigs (purple) versus contigs ≥ 1 kbp (green). (PDF 554 kb) [file 12864_2015_1761_MOESM1_ESM.pdf]

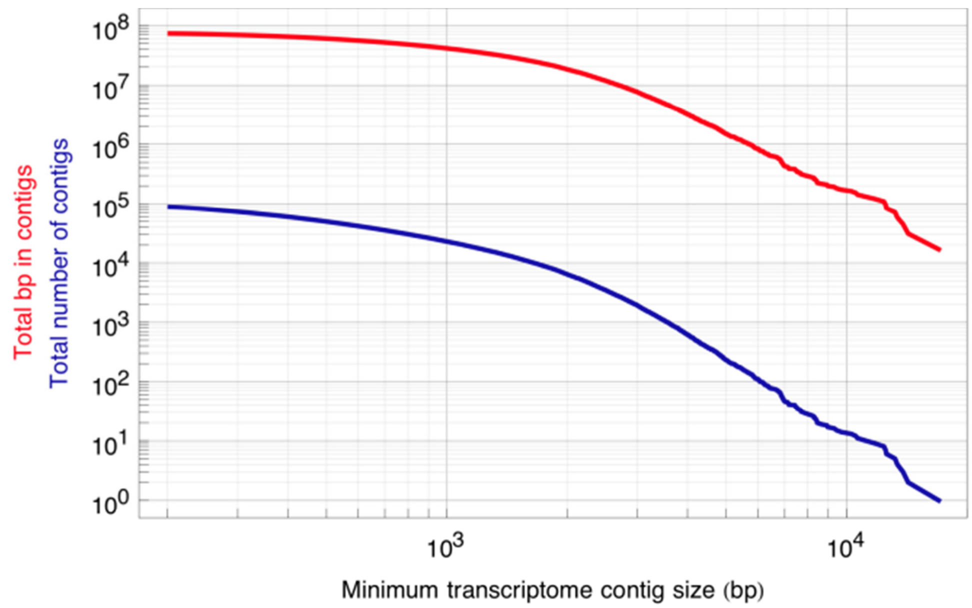

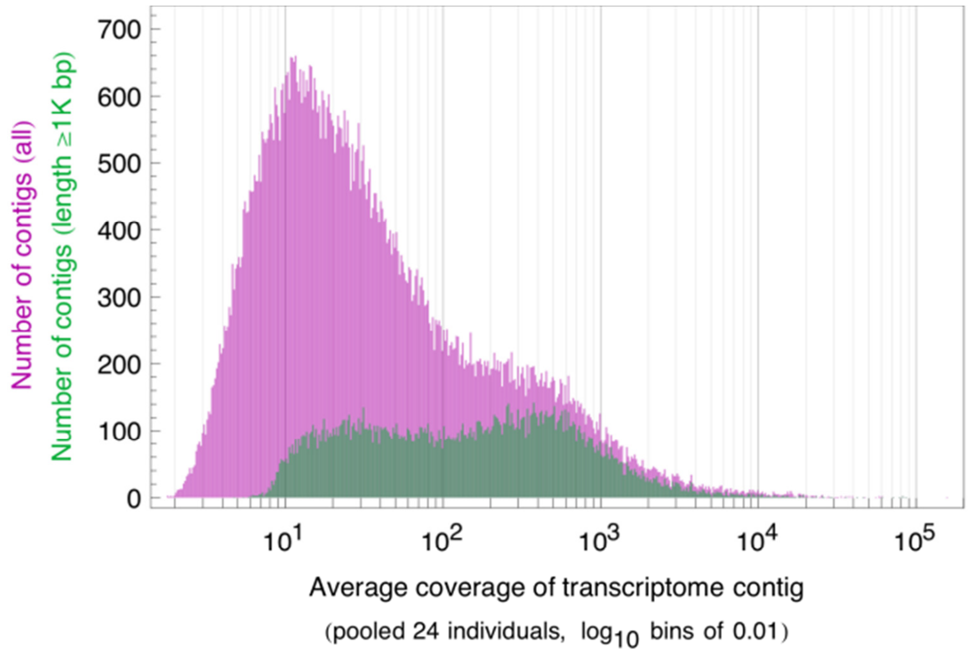

Additional file 1: Transcriptome size and coverage per contig.
(a) Total transcriptome size in base pairs (red) and number of contigs (blue) as minimum contig size threshold in base pairs (*x* axis) is varied; and **(b)** histogram of $\log_{10}$ average coverage for all contigs (purple) versus contigs ≥ 1 kbp (green).
