# Supplementary material for: Evolutionary insights from de novo transcriptome assembly and SNP discovery in California white oaks
Source: BMC Genomics. 2015 Jul 28;16(1):552. doi: 10.1186/s12864-015-1761-4 (PMC4517385; doi:10.1186/s12864-015-1761-4)
Supplement: Additional file 2: — Uniqueness of contigs. Distribution of transcriptome contigs by percent of 100-mers that are ≥ 10 mismatches away from the nearest other 100-mer in the entire transcriptome. (PDF 360 kb) [file 12864_2015_1761_MOESM2_ESM.pdf]

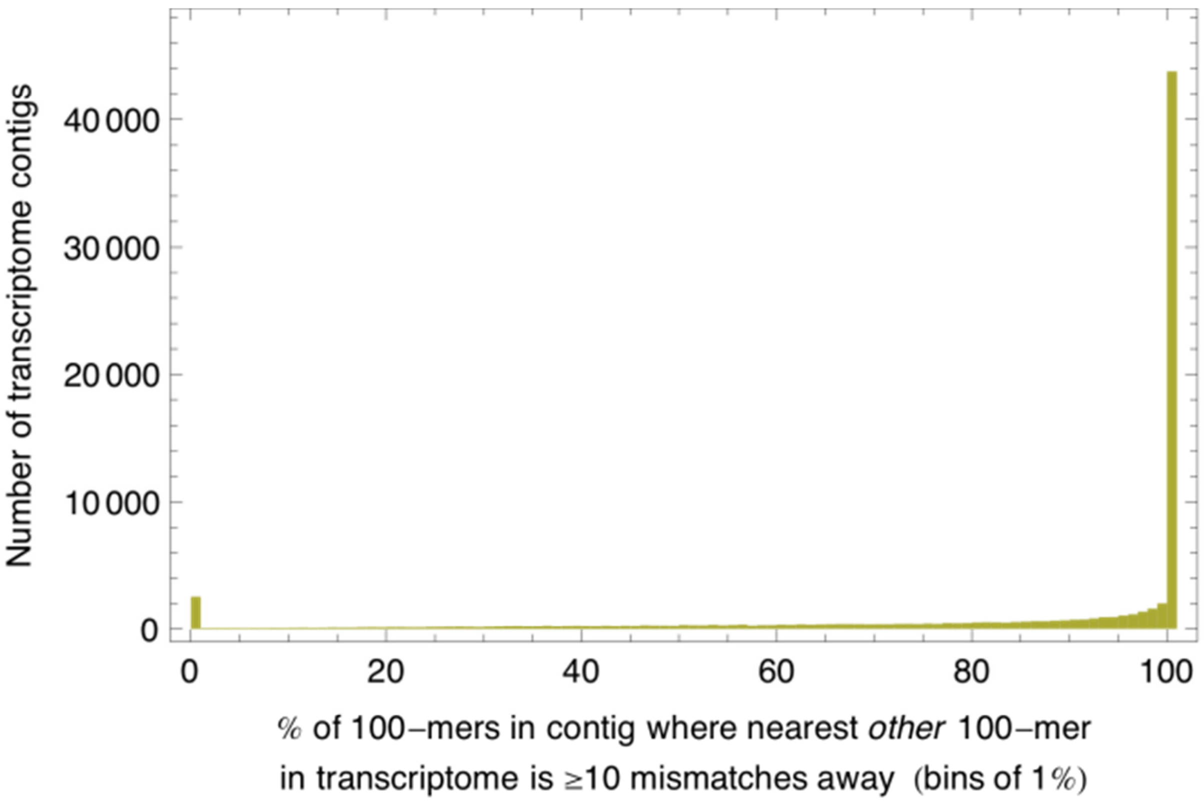

Additional file 2: Uniqueness of contigs.
Distribution of transcriptome contigs by percent of 100-mers that are ≥ 10 mismatches away from the nearest other 100-mer in the entire transcriptome.
