# Supplementary material for: Evolutionary insights from de novo transcriptome assembly and SNP discovery in California white oaks
Source: BMC Genomics. 2015 Jul 28;16(1):552. doi: 10.1186/s12864-015-1761-4 (PMC4517385; doi:10.1186/s12864-015-1761-4)
Supplement: Additional file 3: — C+G content in oak and Arabidopsis . Distribution of C+G content for certain populations of nucleotide sequences (oak solid lines, Arabidopsis dotted lines): coding sequences from all gene models (green, TAIR file TAIR10_cds_20110103_representative_gene_model_updated), 5ʹ-UTRs (blue, TAIR file TAIR10_5_utr_20101028), 3ʹ-UTRs (magenta, TAIR file TAIR10_3_utr_20101028), and entire contigs for oak contigs having no gene models (solid red) or all Arabidopsis intron sequences (dotted red, TAIR file TAIR10_intron_20101028). (PDF 392 kb) [file 12864_2015_1761_MOESM3_ESM.pdf]

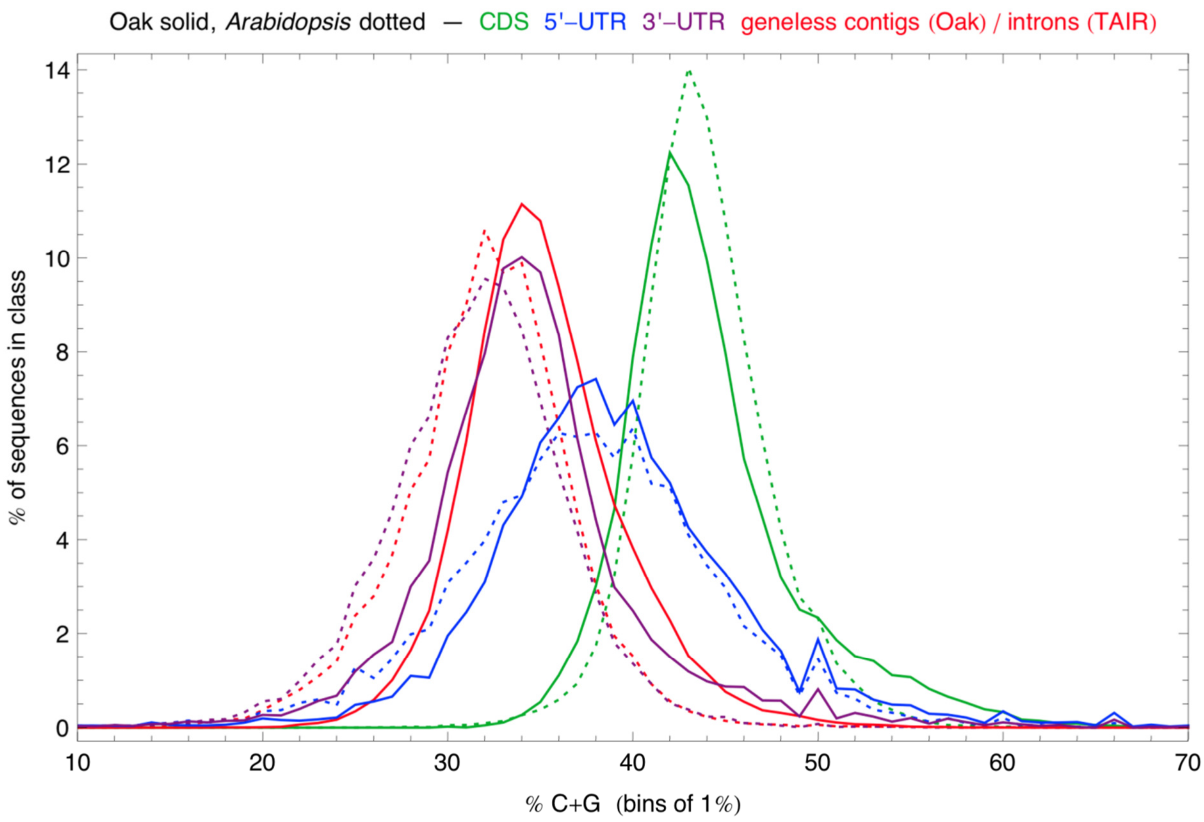

Additional file 3: C+G content in oak and *Arabidopsis*.
Distribution of C+G content for certain populations of nucleotide sequences (oak solid lines, *Arabidopsis* dotted lines): coding sequences from all gene models (green, TAIR file `TAIR10_cds_20110103_representative_gene_model_updated`), 5′-UTRs (blue, TAIR file `TAIR10_5_utr_20101028`), 3′-UTRs (magenta, TAIR file `TAIR10_3_utr_20101028`), and entire contigs for oak contigs having no gene models (solid red) or all *Arabidopsis* intron sequences (dotted red, TAIR file `TAIR10_intron_20101028`).
