# Supplementary material for: Evolutionary insights from de novo transcriptome assembly and SNP discovery in California white oaks
Source: BMC Genomics. 2015 Jul 28;16(1):552. doi: 10.1186/s12864-015-1761-4 (PMC4517385; doi:10.1186/s12864-015-1761-4)
Supplement: Additional file 4: — Overlap in gene content among oak RNA data sets. Pairwise comparison of percentage of transcripts from each transcriptome appearing in the other. (PDF 64 kb) [file 12864_2015_1761_MOESM4_ESM.pdf]

**Database**	***Q. lobata***	***Q. alba* EST**	***Q. robur* EST**
Q. lobata (this work)	—	79%	63%
Q. alba EST	19%	—	32%
Q. robur EST	55%	87%	—

Additional file 4: Overlap in gene content among oak RNA data sets.
Pairwise comparison of percentage of transcripts from each transcriptome appearing in the other.
