# Supplementary material for: Evolutionary insights from de novo transcriptome assembly and SNP discovery in California white oaks
Source: BMC Genomics. 2015 Jul 28;16(1):552. doi: 10.1186/s12864-015-1761-4 (PMC4517385; doi:10.1186/s12864-015-1761-4)
Supplement: Additional file 5: — Comparison of sizes among oak RNA data sets. Comparison of the Quercus lobata transcriptome of this work (all contigs) with the EST-based transcriptomes of Q. alba and Q. robur. (PDF 60 kb) [file 12864_2015_1761_MOESM5_ESM.pdf]

	Number of contigs	Minimum length (bp)	Mean length (bp)	Maximum length (bp)	Total length (Mbp)
Q. lobata (this work)	83,644	203	866	16,982	72.5
Q. alba EST	22,102	73	431	7,401	9.5
Q. robur EST	222,671 (69,154 + 153,517 singletons)	75	426	7,898	94.9

Additional file 5: Comparison of sizes among oak RNA data sets.
Comparison of the *Quercus lobata* transcriptome of this work (all contigs) with the EST-based transcriptomes of *Q. alba* and *Q. robur*.
