# Supplementary material for: Evolutionary insights from de novo transcriptome assembly and SNP discovery in California white oaks
Source: BMC Genomics. 2015 Jul 28;16(1):552. doi: 10.1186/s12864-015-1761-4 (PMC4517385; doi:10.1186/s12864-015-1761-4)
Supplement: Additional file 6: — Comparisons of oak and Arabidopsis amino acid usage and length of gene models and UTRs. (a) Relative frequency of amino acid usage in Arabidopsis TAIR10 gene models compared to complete Quercus gene models ordered left to right from most to least common; and distributions of the length of (b) gene models in amino acids, (c) 5′-UTRs in nucleotides, and (d) 3′-UTRs in nucleotides for Arabidopsis TAIR10 (green) versus complete Quercus models (red). (PDF 763 kb) [file 12864_2015_1761_MOESM6_ESM.pdf]

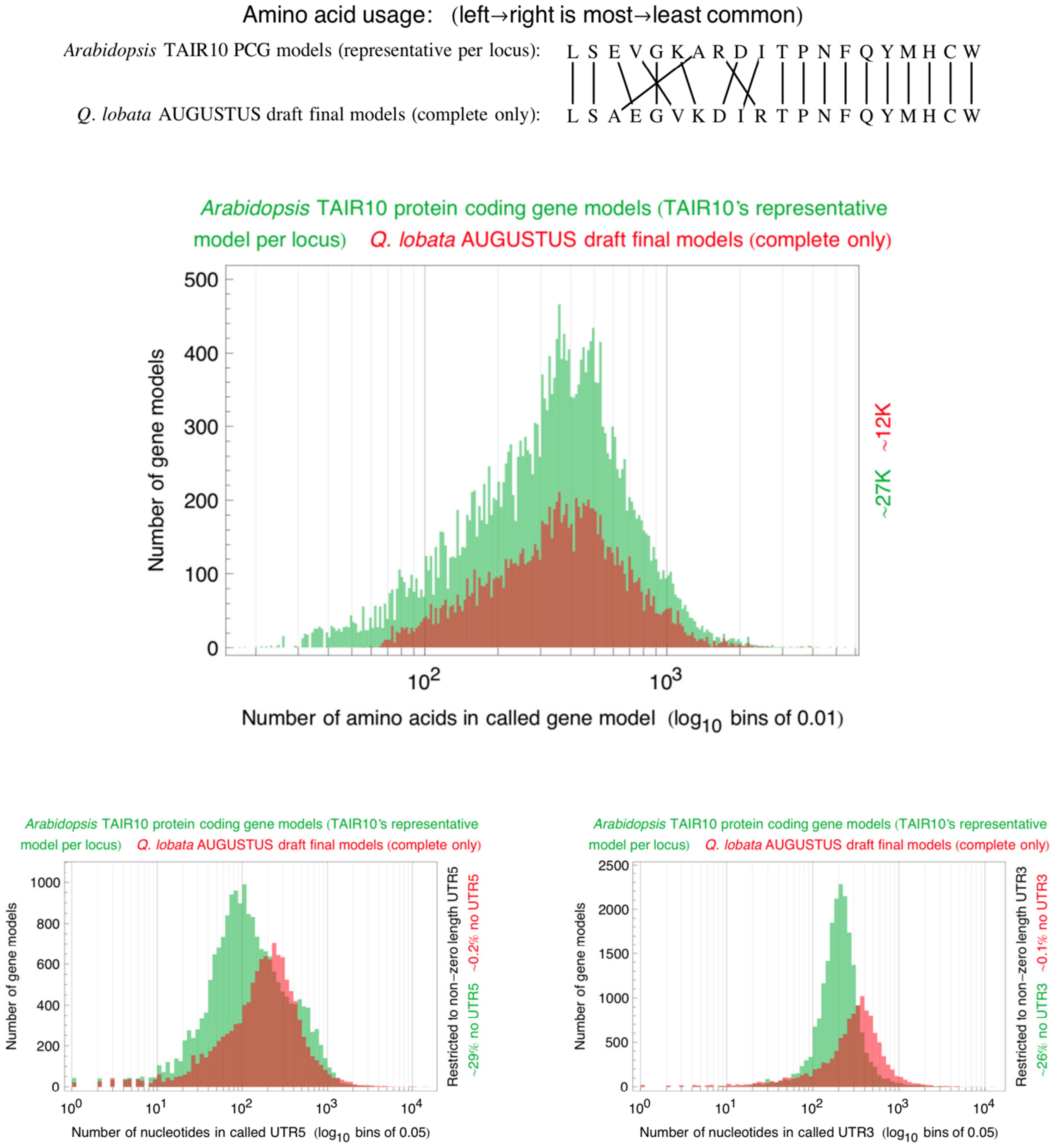

Additional file 6: Comparisons of oak and *Arabidopsis* amino acid usage and length of gene models and UTRs

(a) Relative frequency of amino acid usage in *Arabidopsis* TAIR10 gene models compared to complete *Quercus* gene models ordered left to right from most to least common; and distributions of the length of **(b)** gene models in amino acids, **(c)** 5′-UTRs in nucleotides, and **(d)** 3′-UTRs in nucleotides for *Arabidopsis* TAIR10 (green) versus complete *Quercus* models (red).
