# Supplementary material for: Evolutionary insights from de novo transcriptome assembly and SNP discovery in California white oaks
Source: BMC Genomics. 2015 Jul 28;16(1):552. doi: 10.1186/s12864-015-1761-4 (PMC4517385; doi:10.1186/s12864-015-1761-4)
Supplement: Additional file 7: — Summary of alignments between oak and Arabidopsis orthologs. Histograms of (a) the percent of protein length participating and (b) the percent amino acid identity of amino acid alignments for Arabidopsis–Quercus orthologous genes. (PDF 394 kb) [file 12864_2015_1761_MOESM7_ESM.pdf]

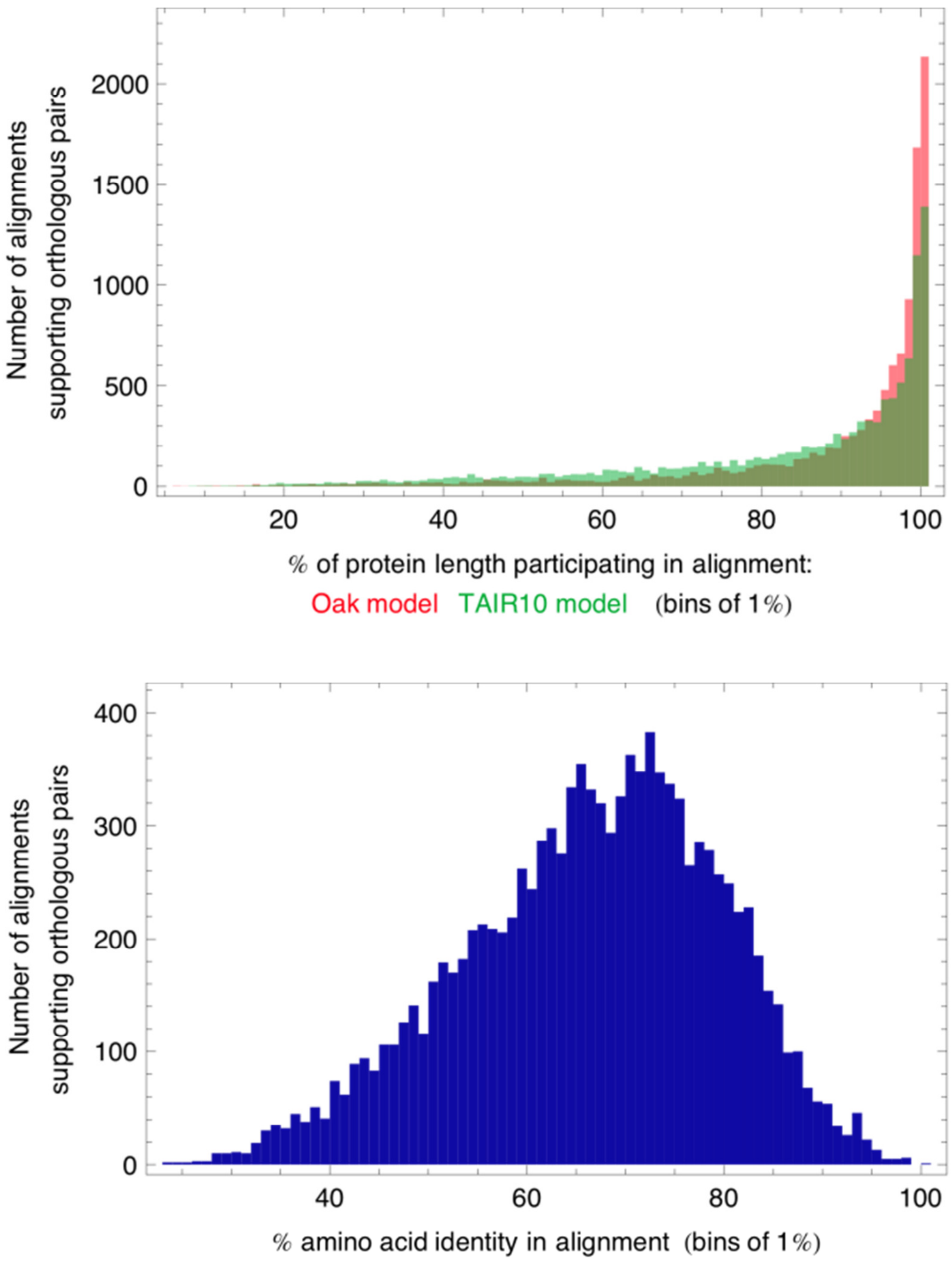

Additional file 7: Summary of alignments between oak and *Arabidopsis* orthologs. Histograms of **(a)** the percent of protein length participating and **(b)** the percent amino acid identity of amino acid alignments for *Arabidopsis–Quercus* orthologous genes.
