# Supplementary material for: Evolutionary insights from de novo transcriptome assembly and SNP discovery in California white oaks
Source: BMC Genomics. 2015 Jul 28;16(1):552. doi: 10.1186/s12864-015-1761-4 (PMC4517385; doi:10.1186/s12864-015-1761-4)
Supplement: Additional file 8: — Comparison of number of and expression level of gene models with and without oak- Arabidopsis orthologous pairing. Histograms by expression level of (a) the number of gene models that are complete and have a called Arabidopsis ortholog (green), partial with an ortholog (yellow), complete without an ortholog (orange), or partial without an ortholog (red); (b) the number of contigs by pooled expression for all contigs (red) versus those with orthologs (green); and (c) the number of Arabidopsis genes by expression levels in a generic Arabidopsis RNA-Seq experiment (NCBI SRX145413 [47], red) versus only those with a called oak ortholog (green). (PDF 559 kb) [file 12864_2015_1761_MOESM8_ESM.pdf]

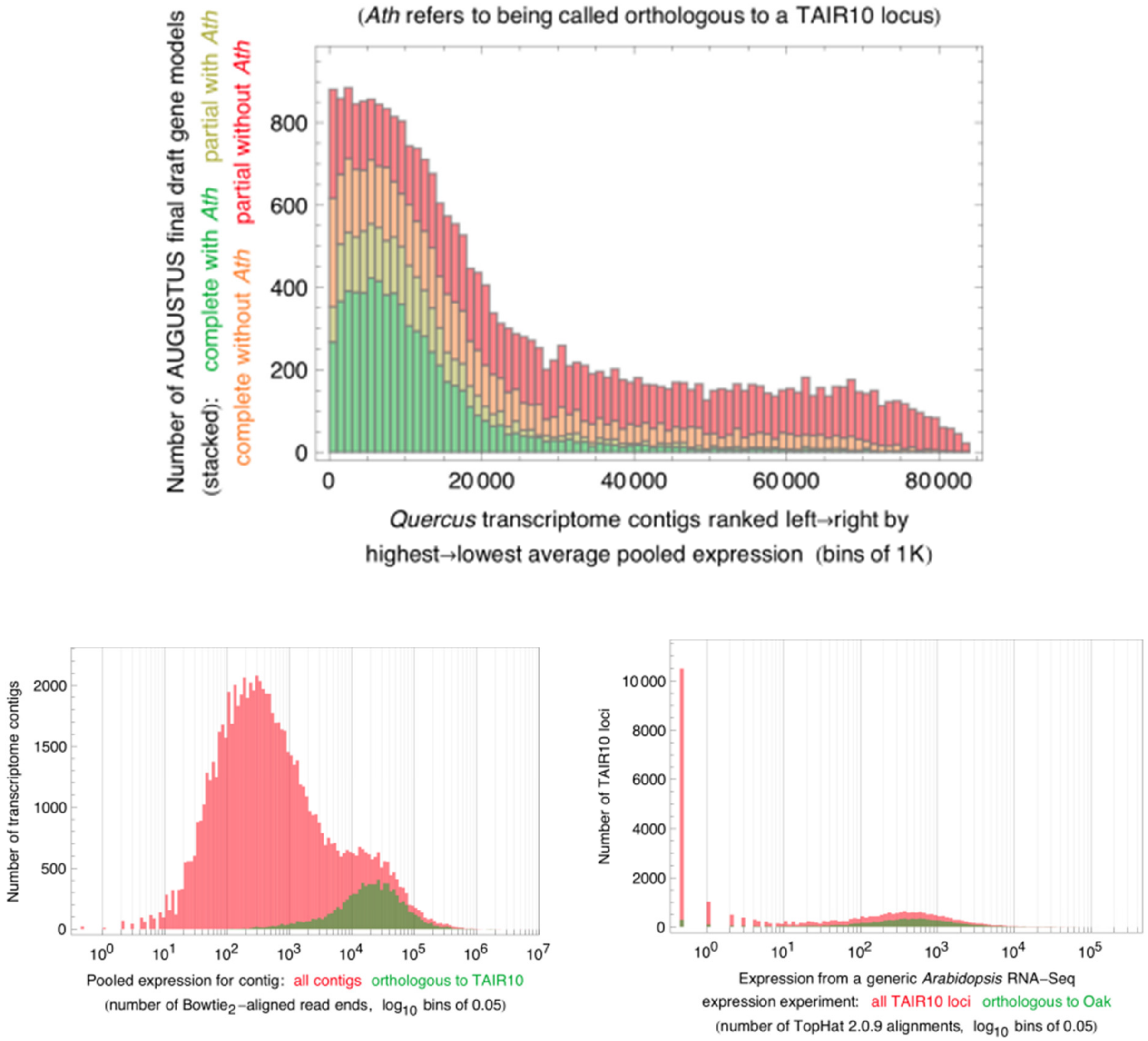

Additional file 8: Comparison of number of and expression level of gene models with and without oak-*Arabidopsis* orthologous pairing.

Histograms by expression level of **(a)** the number of gene models that are complete and have a called *Arabidopsis* ortholog (green), partial with an ortholog (yellow), complete without an ortholog (orange), or partial without an ortholog (red); **(b)** the number of contigs by pooled expression for all contigs (red) versus those with orthologs (green); and **(c)** the number of *Arabidopsis* genes by expression levels in a generic *Arabidopsis* RNA-Seq experiment (NCBI SRX145413 [43], red) versus only those with a called oak ortholog (green).
