# Supplementary material for: Evolutionary insights from de novo transcriptome assembly and SNP discovery in California white oaks
Source: BMC Genomics. 2015 Jul 28;16(1):552. doi: 10.1186/s12864-015-1761-4 (PMC4517385; doi:10.1186/s12864-015-1761-4)
Supplement: Additional file 9: — Expression level in oak versus Arabidopsis orthologs. Two-dimensional histogram of pooled oak expression from oak transcriptome contigs versus expression of the orthologous Arabidopsis gene in a generic Arabidopsis RNA-Seq experiment (NCBI SRX145413 [47]). (PDF 647 kb) [file 12864_2015_1761_MOESM9_ESM.pdf]

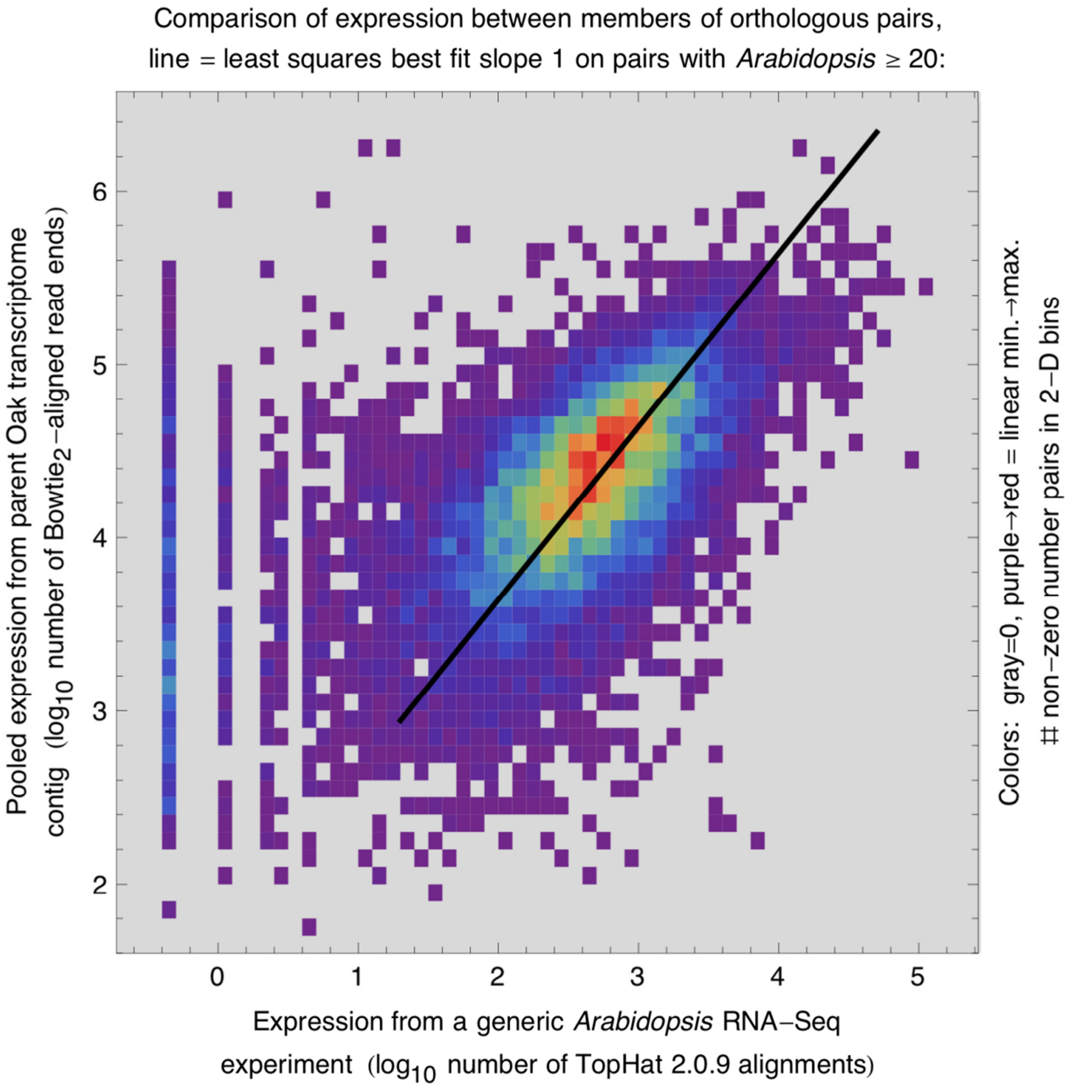

Additional file 9: **Expression level in oak versus *Arabidopsis* orthologs.**
Two-dimensional histogram of pooled oak expression from oak transcriptome contigs versus expression of the orthologous *Arabidopsis* gene in a generic *Arabidopsis* RNA-Seq experiment (NCBI SRX145413 [43]).
