# Supplementary material for: Evolutionary insights from de novo transcriptome assembly and SNP discovery in California white oaks
Source: BMC Genomics. 2015 Jul 28;16(1):552. doi: 10.1186/s12864-015-1761-4 (PMC4517385; doi:10.1186/s12864-015-1761-4)
Supplement: Additional file 10: — Distributions of Gene Ontology terms for oak- Arabidopsis orthologs versus all Arabidopsis genes. Distributions of Gene Ontology Plant Slim functional terms for the Arabidopsis side of the 9,431 Quercus–Arabidopsis ortholog gene pairs (outer rings) versus all Arabidopsis TAIR10 genes (inner ring) for (a) cellular components, (b) biological processes, and (c) molecular functions. (PDF 556 kb) [file 12864_2015_1761_MOESM10_ESM.pdf]

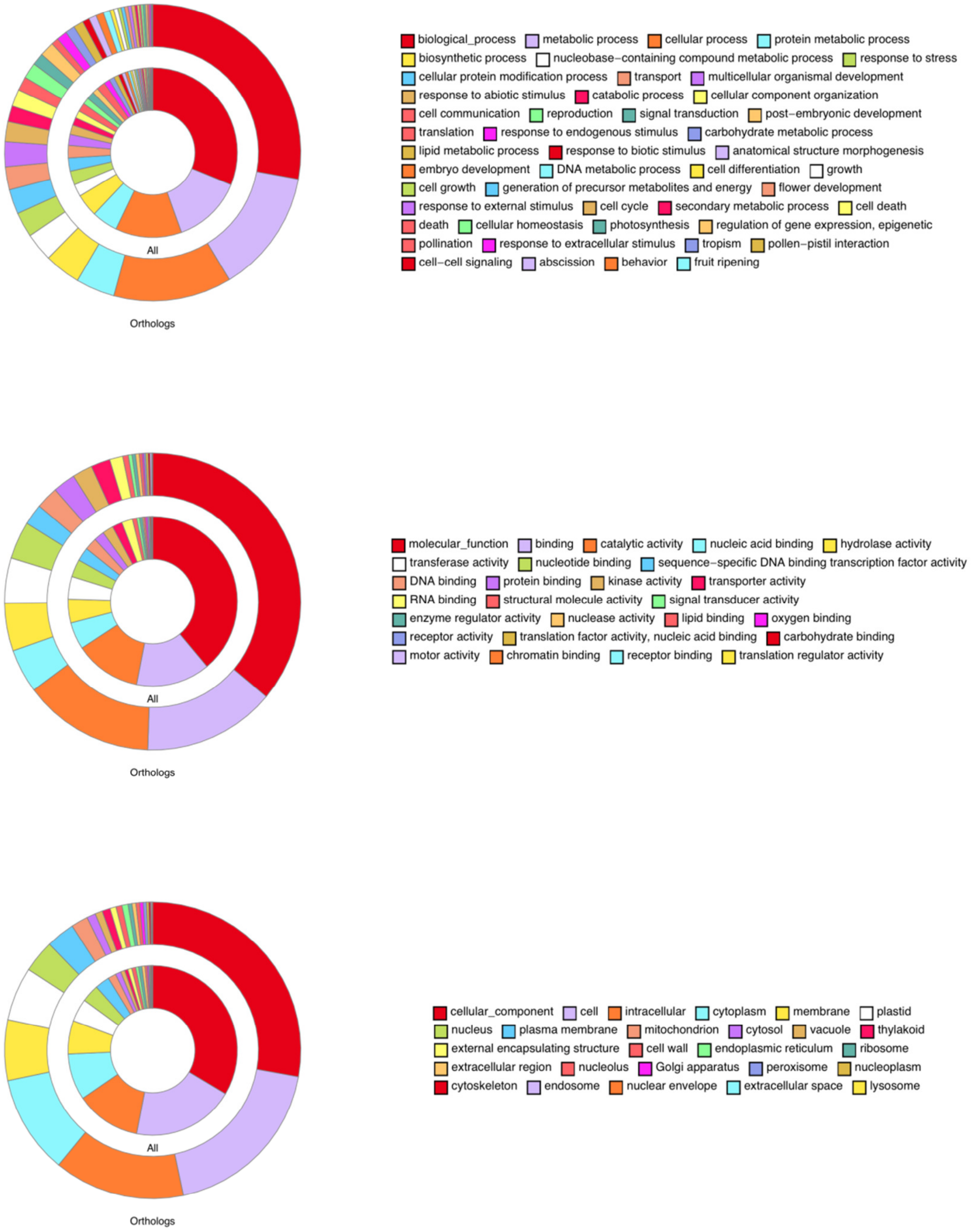

Additional file 10: **Distributions of Gene Ontology terms for oak-*Arabidopsis* orthologs versus all *Arabidopsis* genes.**

Distributions of Gene Ontology Plant Slim functional terms for the *Arabidopsis* side of the 9,431 *Quercus–Arabidopsis* ortholog gene pairs (outer rings) versus all *Arabidopsis* TAIR10 genes (inner ring) for **(a)** cellular components, **(b)** biological processes, and **(c)** molecular functions.
