# Supplementary material for: Evolutionary insights from de novo transcriptome assembly and SNP discovery in California white oaks
Source: BMC Genomics. 2015 Jul 28;16(1):552. doi: 10.1186/s12864-015-1761-4 (PMC4517385; doi:10.1186/s12864-015-1761-4)
Supplement: Additional file 11: — SNP locus rate in oak versus Arabidopsis . Comparison of called SNP locus rate per base pair in (a) Arabidopsis and (b) oak by model-relative position averaged over gene models (stretching/compressing each transcription start to stop span to a nominal length of 2 kbp), including breakdown by coding (green), 5ʹ-UTR (blue), 3ʹ-UTR (light blue), intron (red), and all types combined (black, dashed); and (c) called SNP locus rate per oak base pair as a function of descending coverage, consistent with the low SNP locus rates at the edges of oak in (a) and (b) originating from low coverage at contig edges (and thus lower power to detect variants). (PDF 415 kb) [file 12864_2015_1761_MOESM11_ESM.pdf]

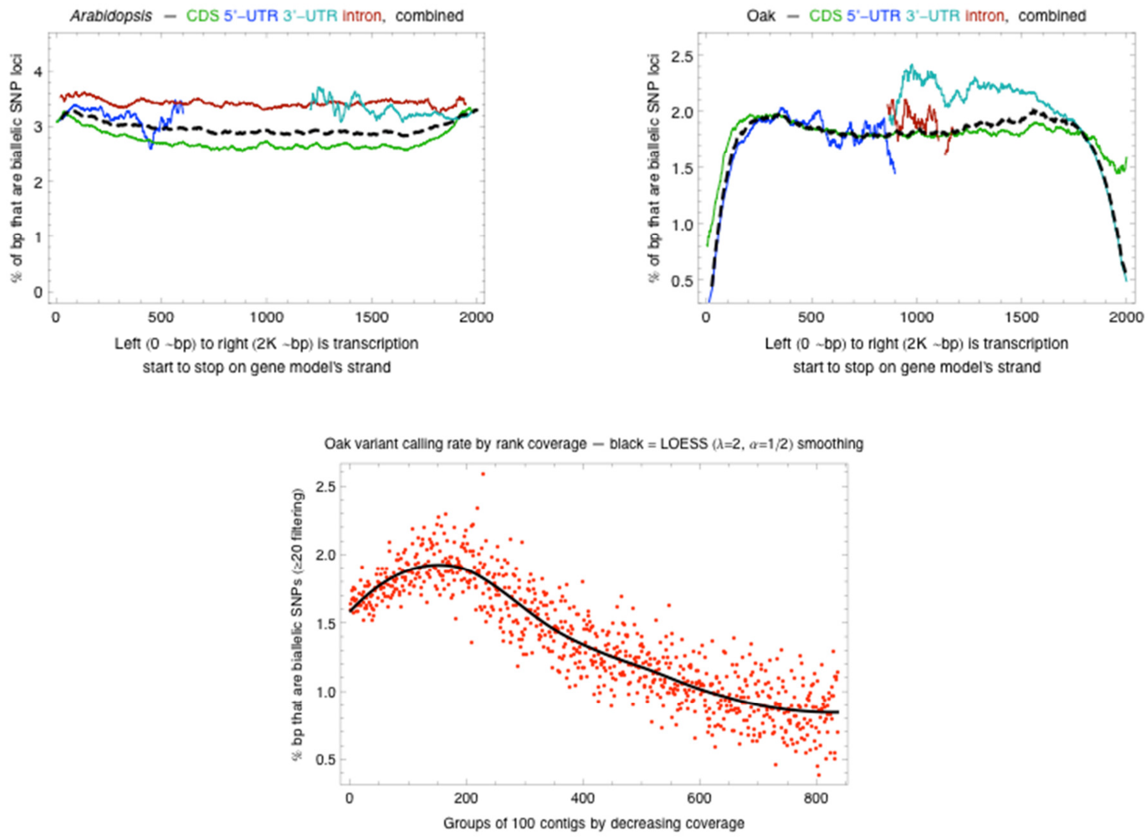

Additional file 11: **SNP locus rate in oak versus *Arabidopsis***.
Comparison of called SNP locus rate per base pair in **(a)** *Arabidopsis* and **(b)** oak by model-relative position averaged over gene models (stretching/compressing each transcription start to stop span to a nominal length of 2 kbp), including breakdown by coding (green), 5′-UTR (blue), 3′-UTR (light blue), intron (red), and all types combined (black, dashed); and **(c)** called SNP locus rate per oak base pair as a function of descending coverage, consistent with the low SNP locus rates at the edges of oak in (a) and (b) originating from low coverage at contig edges (and thus lower power to detect variants).
