# Supplementary material for: Evolutionary insights from de novo transcriptome assembly and SNP discovery in California white oaks
Source: BMC Genomics. 2015 Jul 28;16(1):552. doi: 10.1186/s12864-015-1761-4 (PMC4517385; doi:10.1186/s12864-015-1761-4)
Supplement: Additional file 12: — Pairwise amino acid changes inferred from SNPs inside coding sequence of gene models. Amino acids are given by IUPAC single letter codes (with ‘*’ for STOPs). (PDF 75 kb) [file 12864_2015_1761_MOESM12_ESM.pdf]

	A	C	D	E	F	G	H	I	K	L	M	N	P	Q	R	S	T	V	W	Y	*
**A**	23,742																				
**C**		3,836																			
**D**	1,268		11,243																		
**E**	1,571		7,756	7,545																	
**F**		368			6,237																
**G**	2,582	785	4,096	3,064		16,190															
**H**			1,159				4,875														
**I**					1,220			10,550													
**K**				7,285				560	5,900												
**L**					5,936		1,150	3,853		32,052											
**M**								4,400	1,195	2,441											
**N**			7,094				1,523	945	5,130			8,817									
**P**	1,694						906			4,734			17,944								
**Q**				3,033			3,153		2,084	1,515			974	5,882							
**R**		3,747				3,966	5,744	574	5,027	1,131	627		932	6,037	11,859						
**S**	4,423	2,400			2,483	5,026		1,190		4,811		6,316	5,135		2,790	27,118					
**T**	10,143							6,014	1,542		3,026	1,857	1,692		991	5,863	18,627				
**V**	10,574		1,182	1,775	1,461	2,005		12,365		5,405	3,377							16,664			
**W**		419				227				321					1,951	232					
**Y**		2,243	1,351		2,355		3,061					1,215				1,337				6,101	
*****		85		317	507	90			159	131				902	610	195			505	203	310

Additional file 12: Pairwise amino acid changes inferred from SNPs inside coding sequence of gene models. Amino acids are given by IUPAC single letter codes (with * for STOPs).
