# Supplementary material for: Evolutionary insights from de novo transcriptome assembly and SNP discovery in California white oaks
Source: BMC Genomics. 2015 Jul 28;16(1):552. doi: 10.1186/s12864-015-1761-4 (PMC4517385; doi:10.1186/s12864-015-1761-4)
Supplement: Additional file 13: — Concordance of amino acid change distributions between oak and standard NCBI BLOSUM95. (PDF 331 kb) [file 12864_2015_1761_MOESM13_ESM.pdf]

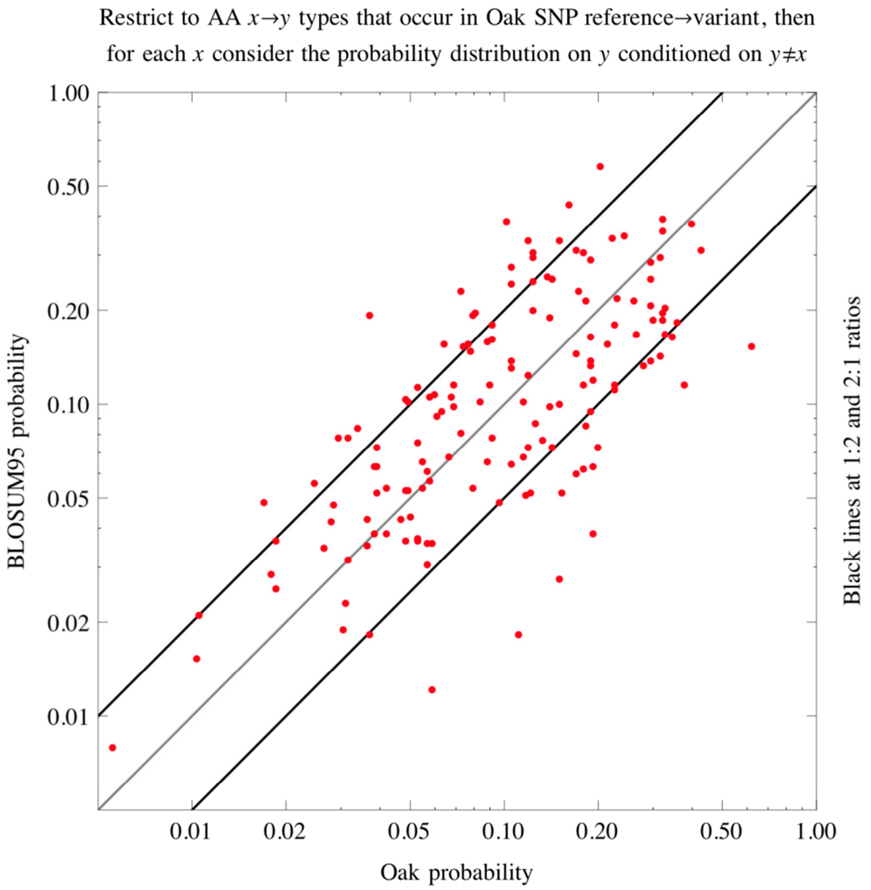

Additional file 13: Concordance of amino acid change distributions between oak and standard NCBI BLOSUM95.
