# Supplementary material for: Evolutionary insights from de novo transcriptome assembly and SNP discovery in California white oaks
Source: BMC Genomics. 2015 Jul 28;16(1):552. doi: 10.1186/s12864-015-1761-4 (PMC4517385; doi:10.1186/s12864-015-1761-4)
Supplement: Additional file 14: — Consistency of called oak genotypes with Hardy-Weinberg equilibrium expectations. Called oak genotypes within Quercus lobata achieve maximum density at black points based on the log posterior density (color gradient). Hardy-Weinberg equilibrium is the white curve. (PDF 351 kb) [file 12864_2015_1761_MOESM14_ESM.pdf]

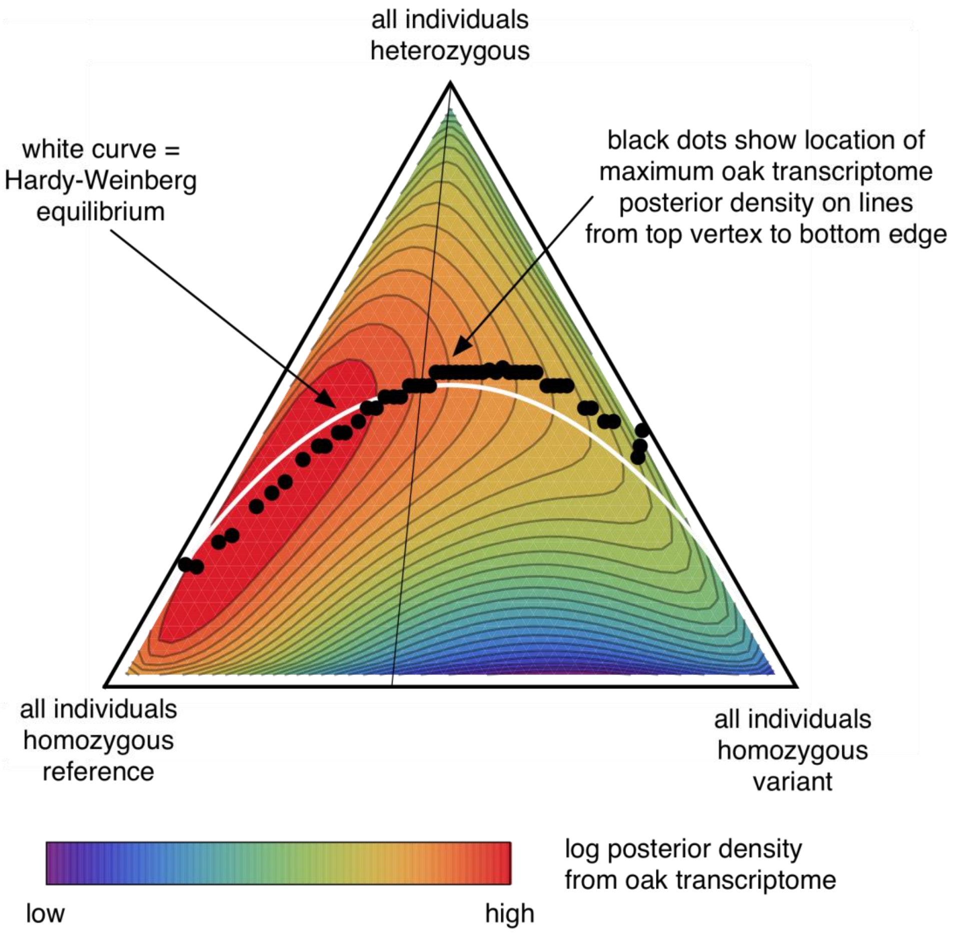

Additional file 14: **Consistency of called oak genotypes with Hardy-Weinberg equilibrium expectations.**

Called oak genotypes within *Quercus lobata* achieve maximum density at black points based on the log posterior density (color gradient). Hardy-Weinberg equilibrium is the white curve.
