# Supplementary material for: Evolutionary insights from de novo transcriptome assembly and SNP discovery in California white oaks
Source: BMC Genomics. 2015 Jul 28;16(1):552. doi: 10.1186/s12864-015-1761-4 (PMC4517385; doi:10.1186/s12864-015-1761-4)
Supplement: Additional file 15: — Histograms of d N / d S ratios and Fay and Wu’s H . (a) d N/d S for Quercus lobata versus Q. garryana (red) and the Q. lobata–Q. douglasii hybrid versus Q. garryana (blue). (b) H for Q. lobata versus its inferred ancestor with Q. garryana. (PDF 225 kb) [file 12864_2015_1761_MOESM15_ESM.pdf]

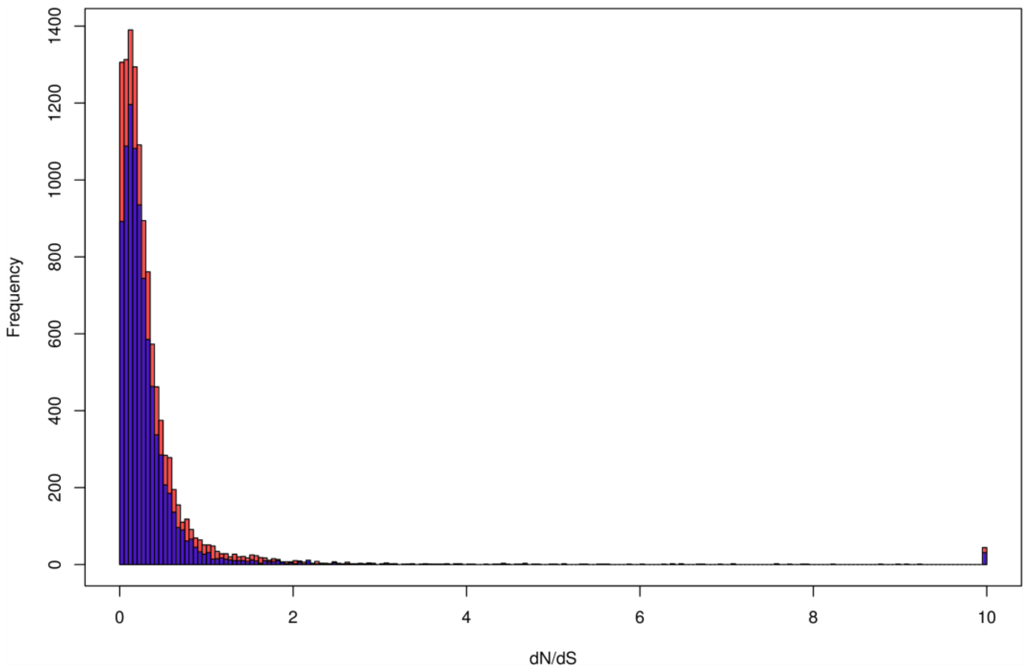


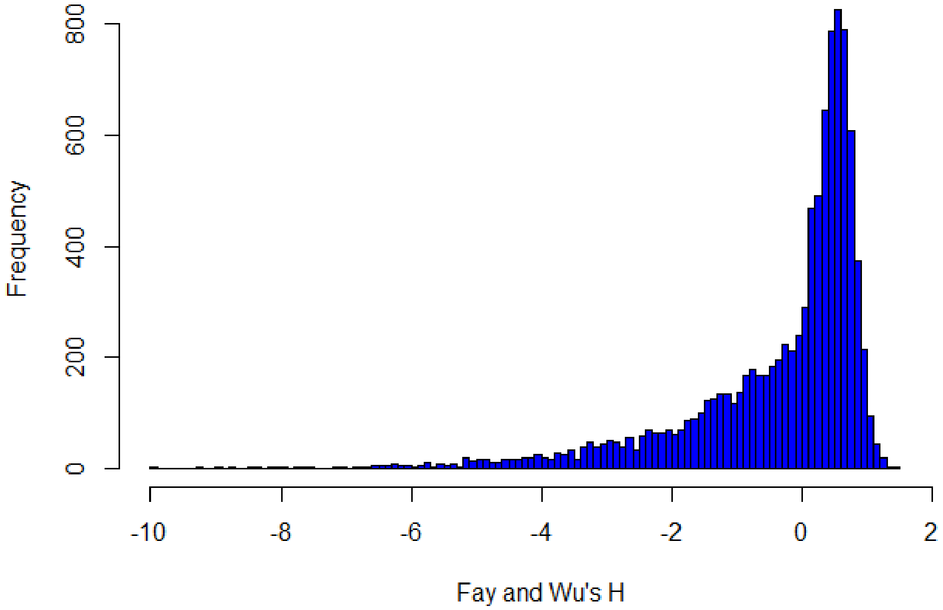


**Additional file 15: Histograms of $d_N/d_S$ ratios and Fay and Wu's *H*.**
**(a)** $d_N/d_S$ for *Quercus lobata* versus *Q. garryana* (red) and the *Q. lobata*–*Q. douglasii* hybrid versus *Q. garryana* (blue). **(b)** *H* for *Q. lobata* versus its inferred ancestor with *Q. garryana*.
