# Supplementary material for: Evolutionary insights from de novo transcriptome assembly and SNP discovery in California white oaks
Source: BMC Genomics. 2015 Jul 28;16(1):552. doi: 10.1186/s12864-015-1761-4 (PMC4517385; doi:10.1186/s12864-015-1761-4)
Supplement: Additional file 21: — SNP evidence for hybrid. (a) Principal components analysis of 226,423 SNPs from 24 Quercus samples, showing the 22 Q. lobata samples (clustered in the the lower right), the Q. garryana individual (in the lower left), and the probable Q. lobata–Q. douglasii hybrid individual (at the top); and (b) proportion of heterozygous genotype calls at SNPs for Q. lobata (blue), Q. garryana (green), and Springville 1 (red, the probable hybrid), with samples ordered left-to-right by latitude south-to-north. (PDF 364 kb) [file 12864_2015_1761_MOESM21_ESM.pdf]

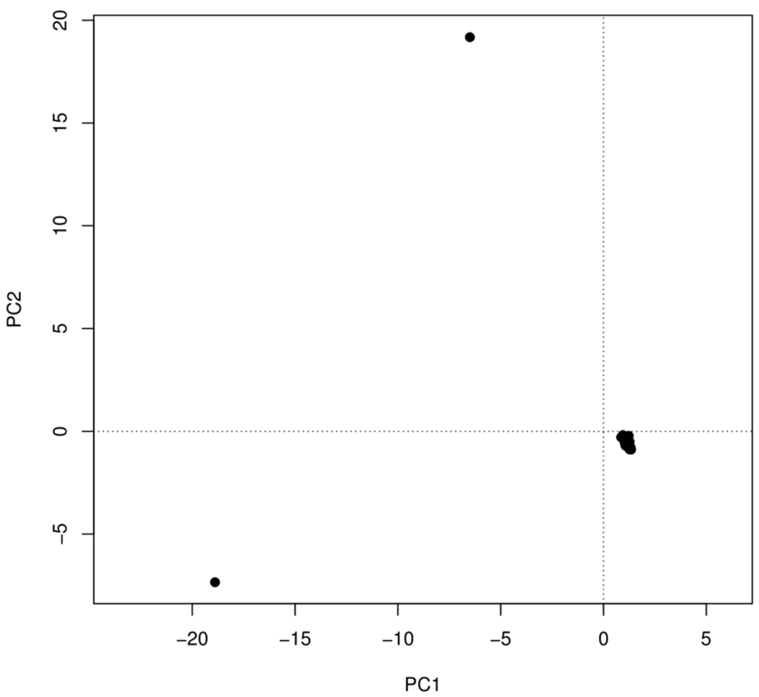

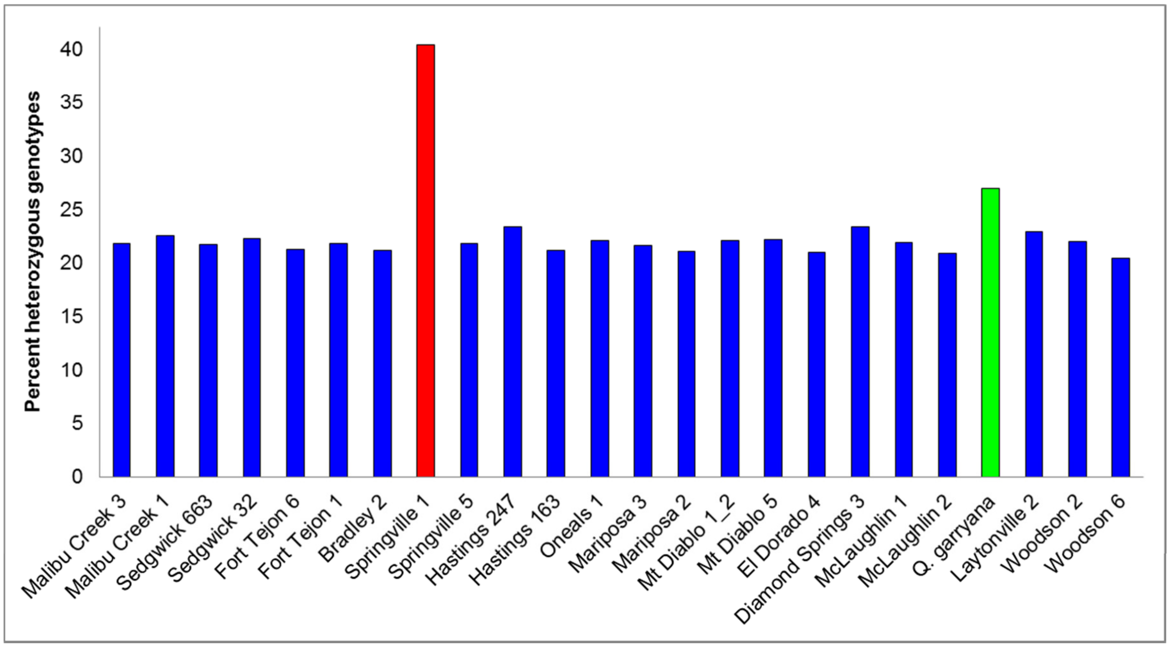

Additional file 19: SNP evidence for hybrid.
(a) Principal components analysis of 226,423 SNPs from 24 *Quercus* samples, showing the 22 *Q. lobata* samples (clustered in the the lower right), the *Q. garryana* individual (in the lower left), and the probable *Q. lobata–Q. douglasii* hybrid individual (at the top); and **(b)** proportion of heterozygous genotype calls at SNPs for *Q. lobata* (blue), *Q. garryana* (green), and Springville 1 (red, the probable hybrid), with samples ordered left-to-right by latitude south-to-north.
